# ATMIN is a transcriptional regulator of both lung morphogenesis and ciliogenesis

**DOI:** 10.1242/dev.107755

**Published:** 2014-10

**Authors:** Paraskevi Goggolidou, Jonathan L. Stevens, Francesco Agueci, Jennifer Keynton, Gabrielle Wheway, Daniel T. Grimes, Saloni H. Patel, Helen Hilton, Stine K. Morthorst, Antonella DiPaolo, Debbie J. Williams, Jeremy Sanderson, Svetlana V. Khoronenkova, Nicola Powles-Glover, Alexander Ermakov, Chris T. Esapa, Rosario Romero, Grigory L. Dianov, James Briscoe, Colin A. Johnson, Lotte B. Pedersen, Dominic P. Norris

**Affiliations:** 1 Mammalian Genetics Unit, MRC Harwell, Harwell Science and Innovation Campus, Oxfordshire OX11 0RD, UK; 2 Section of Ophthalmology and Neurosciences, Wellcome Trust Brenner Building, Leeds Institute of Molecular Medicine, St James's University Hospital, Beckett Street, Leeds LS9 7TF, UK; 3 Department of Biology, University of Copenhagen, Universitetsparken 13, Copenhagen, OE DK-2100, Denmark; 4 Cancer Research UK and Medical Research Council Oxford Institute for Radiation Oncology, Department of Oncology, University of Oxford, Oxford OX3 7DQ, UK; 5 Department of Chemistry, Lomonosov Moscow State University, Leninskie Gory 1-11, Moscow 119991, Russia; 6 MRC National Institute for Medical Research, Mill Hill, London NW7 1AA, UK; 7 MRC National Institute for Medical Research, Mill Hill, London NW7 1AA, UK

**Keywords:** Asciz, Atmin, Ciliogenesis, Ciliopathy, *Dynll1*, Mouse

## Abstract

Initially identified in DNA damage repair, ATM-interactor (ATMIN) further functions as a transcriptional regulator of lung morphogenesis. Here we analyse three mouse mutants, *Atmin^gpg6/gpg6^*, *Atmin^H210Q/H210Q^* and *Dynll1^GT/GT^*, revealing how ATMIN and its transcriptional target dynein light chain LC8-type 1 (DYNLL1) are required for normal lung morphogenesis and ciliogenesis. Expression screening of ciliogenic genes confirmed *Dynll1* to be controlled by ATMIN and further revealed moderately altered expression of known intraflagellar transport (IFT) protein-encoding loci in *Atmin* mutant embryos. Significantly, *Dynll1^GT/GT^* embryonic cilia exhibited shortening and bulging, highly similar to the characterised retrograde IFT phenotype of *Dync2h1.* Depletion of ATMIN or DYNLL1 in cultured cells recapitulated the *in vivo* ciliogenesis phenotypes and expression of DYNLL1 or the related DYNLL2 rescued the effects of loss of ATMIN, demonstrating that ATMIN primarily promotes ciliogenesis by regulating *Dynll1* expression. Furthermore, DYNLL1 as well as DYNLL2 localised to cilia in puncta, consistent with IFT particles, and physically interacted with WDR34, a mammalian homologue of the *Chlamydomonas* cytoplasmic dynein 2 intermediate chain that also localised to the cilium. This study extends the established *Atmin-Dynll1* relationship into a developmental and a ciliary context, uncovering a novel series of interactions between DYNLL1, WDR34 and ATMIN. This identifies potential novel components of cytoplasmic dynein 2 and furthermore provides fresh insights into the molecular pathogenesis of human skeletal ciliopathies.

## INTRODUCTION

Cilia are membrane bounded microtubule-based extensions of the centrosome that demonstrate varied roles in mammalian development and adult physiology (Satir and Christensen, 2007; Quinlan et al., 2008; Baker and Beales, 2009; Norris and Grimes, 2012). Motile cilia are requisite to normal respiratory and reproductive tract function, left-right (L-R) patterning and ependymal cell function (Quinlan et al., 2008). Immotile primary cilia, present on most cells, are involved in wide-ranging functions that include cell-cell signalling, force perception, sensation and cell polarity (Satir and Christensen, 2007; Baker and Beales, 2009; Oh and Katsanis, 2012).

Proteomic, genomic and genetic analyses have revealed hundreds of proteins to be present in cilia (Gherman et al., 2006). These are transported into and out of cilia by a process termed intraflagellar transport (IFT) (Kozminski et al., 1993). IFT particles moving toward the ciliary tip (anterograde) and those moving back towards the cell (retrograde) are driven by kinesin 2 and cytoplasmic dynein 2 motors, respectively (Rosenbaum and Witman, 2002; Pedersen and Rosenbaum, 2008). Whereas the components of kinesin 2 are well known, the constitution of cytoplasmic dynein 2 remains uncertain (Pfister et al., 2006). In comparison to six proteins identified as comprising cytoplasmic dynein 1, only two proteins (DYNC2H1 and DYNC2LI1) have been definitively identified for mammalian cytoplasmic dynein 2 (Pfister et al., 2006). In *Chlamydomonas reinhardtii*, the cytoplasmic dynein 2 complex is known to contain at least five different subunits (Rompolas et al., 2007; Patel-King et al., 2013), suggesting that other components remain to be identified for mammalian cytoplasmic dynein 2. In addition to motors and cargo, IFT particles also comprise IFT proteins that organise into two different complexes, IFT-A and IFT-B, with roles in anterograde and retrograde transport as well as ciliary trafficking of membrane proteins (Goetz and Anderson, 2010; Pedersen and Christensen, 2012). Mutations in IFT or motor protein that prevent anterograde transport lead to loss of overt cilia, whereas those affecting retrograde IFT are associated with shortened and often malformed cilia (Pedersen and Rosenbaum, 2008; Goetz and Anderson, 2010).

The crucial role of both motile and immotile (primary) cilia in mammalian development and disease have become increasingly evident (Satir and Christensen, 2007; Quinlan et al., 2008; Baker and Beales, 2009; Norris and Grimes, 2012). Complete loss of cilia results in early embryonic lethality (Norris and Grimes, 2012), whereas postnatal cilia loss leads to obesity and polycystic kidney disease (Davenport et al., 2007). A variety of cilia-based defects underlie both embryonic lethal and adult viable human syndromes, including Jeune syndrome (JS), Meckel-Grüber syndrome (MKS), Joubert syndrome (JBTS), short-rib polydactyly syndrome (SRPS), Bardet-Biedl syndrome (BBS) and Alström syndrome (ALS). Collectively these are known as the ciliopathies (Badano et al., 2006). Initially defined through the overlapping signs and symptoms of a small collection of disorders (Badano et al., 2006), this group has expanded, and continues to expand, through systematic analysis of overlapping syndromes (Baker and Beales, 2009) and model organism genetics (Norris and Grimes, 2012). Intriguingly, individual genes can underlie multiple ciliopathies; variations in allele strength determine the precise syndromic outcome (Hildebrandt et al., 2011). However, evidence has also been advanced for multilocus-driven ciliopathies (Burghes et al., 2001; Katsanis et al., 2001), and it is easy to see how an allele at a second locus could influence the severity of a ciliopathy. Indeed, studies in mouse have revealed how heterozygous mutations in IFT loci can influence the severity of *Dync2h1* mutants, acting to suppress aspects of the phenotype (Ocbina et al., 2011). While many of the genes involved in ciliogenesis remain to be identified, it is evident that ciliary defects underlie a significant number of developmental human disorders (Baker and Beales, 2009).

Although a core set of ciliopathy signs and symptoms has been defined, these are not present in every ciliopathy. Indeed, rarer defects are evident in only a proportion of ciliopathies, while severe developmental defects incompatible with life will *a priori* occur only in lethal ciliopathies. Deficiencies in pulmonary development and patterning have been reported for a subset of ciliopathies: pulmonary hypoplasia is described for some SRPS (MIM: 263520) and JBTS (MIM: 208500) patients. The lethal ciliopathy hydrolethalus syndrome (MIM: 236680) has been reported to result in pulmonary agenesis (Toriello and Bauserman, 1985; Dammermann et al., 2009). A mouse model of MKS demonstrates pulmonary hypoplasia (Weatherbee et al., 2009), whereas the *Wrd35^yeti^* mutant, a SRPS model, develops lung hypoplasia with tracheal-esophageal fistula (Mill et al., 2011). Hypomorphic mutations in two IFT loci also lead to pulmonary aplasia/hypoplasia: *Ift172^avc^* mutants exhibit pulmonary aplasia (Huangfu and Anderson, 2006); *Ift88^cbs^* leads to incidence of aplasia and hypoplasia (Willaredt et al., 2008). We have previously reported identifying a class of lethal mouse ciliopathy models that exhibit pulmonary hypoplasia and/or agenesis (Ermakov et al., 2009). Together, these data support the argument that pulmonary defects can result from cilia dysfunction; we were therefore intrigued by the reported lung phenotype of a mouse mutant in ATM-interactor (ATMIN; also known as ASCIZ) (Jurado et al., 2010).

Initially identified as a DNA damage response protein (McNees et al., 2005) involved in base excision repair and *in vivo* oxidative stress responses (Jurado et al., 2010; Kanu et al., 2010), ATMIN was subsequently revealed to also function as a zinc finger (ZF)-containing transcription factor regulating embryonic lung development; lungs and trachea are absent from *Atmin*^−/−^ embryos (Jurado et al., 2010). Analysis of the transcriptional activity of ATMIN has revealed it to directly regulate the *Dynll1* dynein light chain locus (Jurado et al., 2012a). The two proteins directly interact such that DYNLL1 protein binds to ATMIN, reducing its transcriptional activity. In light of the *Atmin* phenotype and the known role of *Chlamydomonas* LC8 (a *Dynll1* homologue) in ciliogenesis (Pazour et al., 1998), we hypothesised that ATMIN regulates ciliogenesis.

We identified two mouse point mutants (*Atmin^gpg6^* and *Atmin^H210Q^*) in the ATMIN ZFs. Both resulted in an embryonic lethal phenotype highly similar to that of the null mutant (*Atmin*^−^) and reminiscent of a ciliopathy. Indeed, analysis of embryonic nodes, limb buds and neural tubes revealed shortened cilia in *Atmin* mutant embryos. ATMIN zinc fingers are required for transcription factor activity (Jurado et al., 2012b); analysis of embryonic gene expression revealed modest, but consistent, reductions in *FoxJ1*, *Ift88*, *Ift172* and *Ift140* expression in the mutants. As expected, a highly significant drop in *Dynll1* expression was also detected, although its close paralogue *Dynll2* remained unaffected. Analysis of *Dynll1* mutant embryos revealed a gross embryonic phenotype highly similar to *Atmin*. Shortened nodal cilia were evident and demonstrated a bulging morphology highly reminiscent of cytoplasmic dynein 2 mutants. Knockdown of *Atmin* or *Dynll1* in cultured cells resulted in almost identical phenotypes; fewer ciliated cells and reduced ciliary length, a phenotype rescued by overexpression of DYNLL1 or DYNLL2. Both DYNLL1 and DYNLL2 exhibited ciliary localised puncta, consistent with IFT particles, as might be predicted for cytoplasmic dynein 2. Analysis of hedgehog signalling revealed this to be downregulated tissue specifically in the developing lungs. We further demonstrated the putative dynein intermediate chain, WDR34, to localise to cilia and to directly interact with DYNLL1. Together, these data support the argument that ATMIN regulates DYNLL1, which in turn controls retrograde IFT and ciliogenesis. Thus, these data provide evidence of an ATMIN-DYNLL1 pathway controlling ciliogenesis.

## RESULTS

### ATMIN function is required for normal development

We identified the gasping mutants as demonstrating ciliopathy-like phenotypes and defective cilia in a forward genetic screen (Ermakov et al., 2009). Haplotypic analysis and candidate gene sequencing of gasping 6 (*gpg6*) revealed a T to A transversion in exon 3 of *Atmin*, correlating with the third ZF (supplementary material Fig. S1A,B). The resulting cysteine to serine substitution in the fourth canonical residue associated with Zn^2+^ chelation alters a highly conserved residue within a conserved region of the protein (supplementary material Fig. S1A,D). Mutation of ZF canonical residues is known to destroy function; indeed, an equivalent mutation in *Zic2* has been specifically demonstrated to abolish DNA binding (Brown et al., 2005). Utilising published anti-ATMIN antibodies (McNees et al., 2005), we assessed ATMIN protein in mutant and wild-type embryonic tissue. Multiple bands were evident on western blots (supplementary material Fig. S2A), including a pair of bands at the published size (supplementary material Fig. S2B); a pair of ATMIN bands have previously been reported (McNees et al., 2005). The lower of these bands was undetectable in the mutant samples; however, no change in the intensity of the higher band was evident (supplementary material Fig. S2B). It seems highly probable that ATMIN^gpg6^ protein will not function as a transcription factor, but we are unable to rule out the impact of altered levels of post-translational modification.

**Fig. 1.**
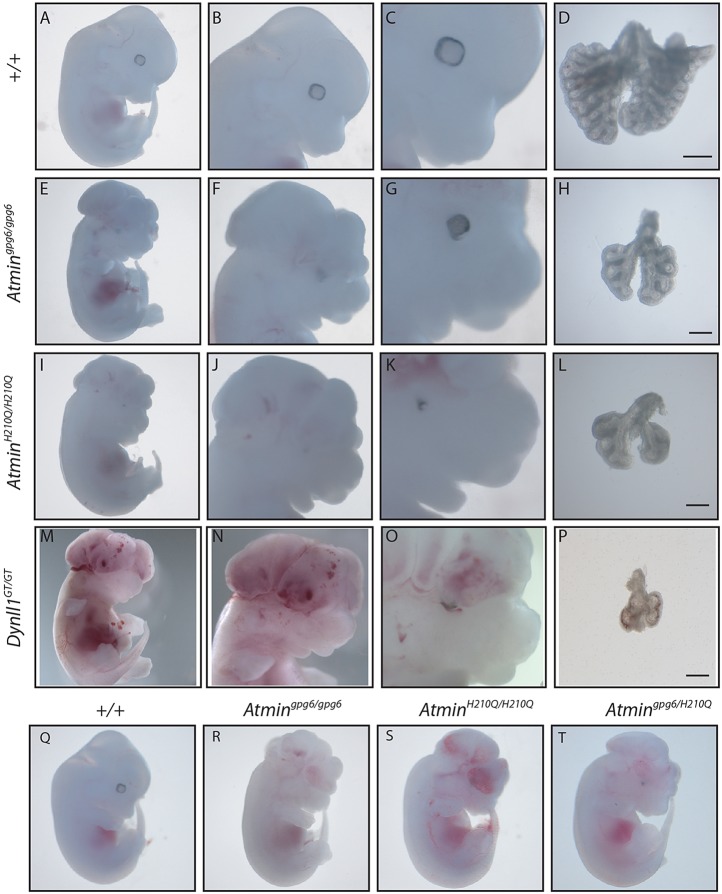
***Atmin* and *Dynll1* mutants show characteristics of a ciliopathy.** Wild-type 13.5 dpc embryo (A), showing normal head formation (B) and a ring of pigmented retinal epithelium surrounding the eye (C). Correctly patterned lungs show a single left- and four right-sided lobes (D). *Atmin^gpg6^*^/*gpg6*^ 13.5 dpc mutant embryo (E), showing exencephaly (F) and abnormal pigmented retinal epithelium distribution (G). The lungs are small and demonstrate left isomerism (H). *Atmin^H210Q/H210Q^* 13.5 dpc mutant embryo (I) demonstrating exencephaly (J), micropthalmia (K) and small lungs with left isomerism (L). *Atmin^H210Q^*^/+^×*Atmin^gpg6/+^* intercrosses give rise to wild-type (Q) and *Atmin^gpg6/H210Q^* (T) embryos, that phenocopy *Atmin^gpg6^*^/*gpg6*^ (R) and *Atmin^H210Q^*^/*H210Q*^ (S) embryos. *Dynll1^GT/GT^* 13.5 dpc embryos (M) also display exencephaly (N), abnormal retinal epithelium distribution (O) and very small, mispatterned lungs (P). Scale bars: 1 mm in D; 200 μm in H,L,P.

In order to confirm our previous findings (Ermakov et al., 2009), a congenic C3.C-*gpg6* strain was generated, segregating away additional mutations and providing a defined, homogeneous genetic background. Analysis of homozygous C3.C-*gpg6* embryos revealed a similar gross phenotype to that we previously reported: combinations of oedema, exencephaly, pulmonary hypoplasia and pulmonary situs anomalies (Table 1; Fig. 1E-H). However, in contrast to the variability of lung size (full sized, small or absent) seen in the outbred stock, only small lungs were evident (Fig. 1H), all of which demonstrated abnormal situs (Table 1). The low level micrognathia we previously reported was no longer obvious, but craniofacial defects remained evident, most obviously in the form of a thickening of the philtrum (Table 1). Both cardiac and gut situs were wild type, but abnormal cardiac outflow tract development was evident in almost all embryos (supplementary material Table S1).

**Table 1.**
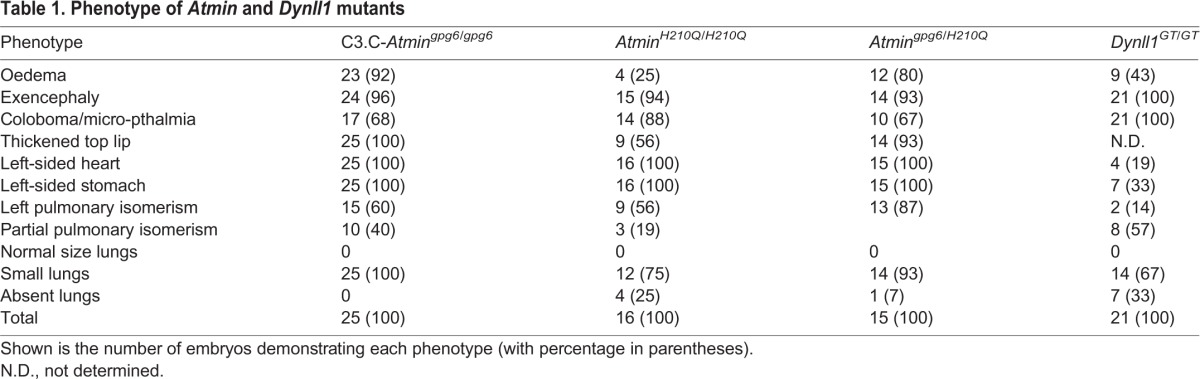
Phenotype of *Atmin* and *Dynll1* mutants

*Atmin* has a well-characterised role in DNA damage pathways (Kanu and Behrens, 2008). In order to assess whether *Atmin^gpg6^*^/*gpg6*^ embryonic phenotypes might relate to a deficiency in the DNA damage response, we analysed the intracellular localisation of the DNA damage marker 53BP1 in wild-type (supplementary material Fig. S3A) and mutant embryos (supplementary material Fig. S3B). Relocalisation of 53BP1 into discrete nuclear foci is an accepted indicator of genome instability (Schultz et al., 2000; Wang et al., 2002) and was readily detected in wild-type embryos treated with an alkylating DNA damaging agent, MMS (supplementary material Fig. S3C). However, we were unable to detect any 53BP1 foci formation in mutant embryos, consistent with arguments that the impact of ATMIN on embryogenesis is independent from its role in DNA damage (Heierhorst et al., 2011).

A second allele (*Atmin^H210Q^*) was identified through reverse genetic screening of an ENU-mutated DNA archive (Quwailid et al., 2004) (supplementary material Fig. S1A,C); *Atmin^H210Q^* contains a histidine to glutamine substitution predicted to destroy function of the fourth ZF, again by interfering with Zn^2+^ chelation. Phenotypic analysis of *Atmin^H210Q^*^/*H210Q*^ embryos revealed highly similar phenotypes to *Atmin^gpg6^*^/*gpg6*^ (Fig. 1I-L and Table 1). Notably, lungs were absent from 25% of these embryos, similar to the original outbred *Atmin^gpg6^*^/*gpg6*^ mutants (Ermakov et al., 2009), most likely reflecting the non-inbred nature of *Atmin^H210Q^*. Genetic non-complementation was evident between the two *Atmin* mutants; all *Atmin^gpg6^*^/*H210Q*^ embryos analysed showed an identical phenotype, highly similar to that of the individual homozygous mutations (Fig. 1Q-T and Table 1), confirming that the *Atmin* mutation underlies the *gpg6* phenotype.

### ATMIN is required for correct cilia length in the embryo

**Fig. 2.**
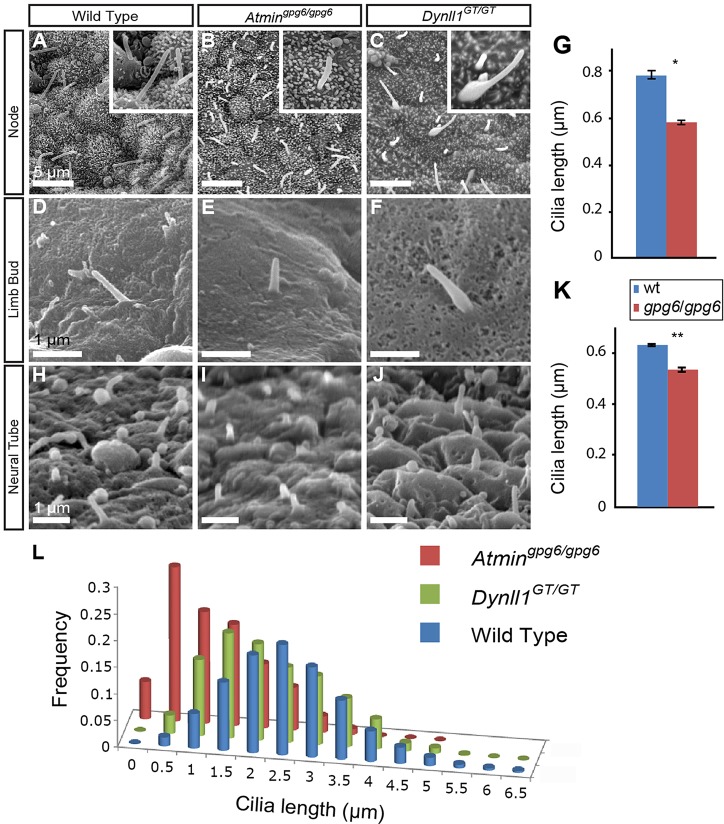
**ATMIN and DYNLL1 are required for normal ciliogenesis.** Tissue from stage-matched wild-type (A,D,H), *Atmin^gpg6^*^/*gpg6*^ (B,E,I) and *Dynll1^GT/GT^* (C,F,J) embryos imaged by SEM. (A-C) Two- to four-somite stage embryonic nodes; insets show higher magnification of one cilium from the main panel. (D-F) Limb bud cilia at 11.5 dpc. (H-J) Neural tube cilia at 11.5 dpc. (G) Comparative analysis of limb bud cilia length (*n*∼75, three embryos per class). (K) Comparative analysis of neural tube cilia length (*n*∼450, three embryos per class). *P=0.027, **P=0.0106. *Dynll1^GT/GT^* cilia width was substantially increased in both limb bud (F) and neural tube (J) (see supplementary material Fig. S7 for statistical analysis). (L) Distribution of nodal cilia length is shifted towards shorter cilia in *Atmin^gpg6^*^/*gpg6*^ and *Dynll1^GT/GT^* mutants (*n*∼500, three embryos per class).

Initial characterisation of *gpg6* had demonstrated short, stumpy nodal cilia (Ermakov et al., 2009). However, congenic C3.C-*Atmin^gpg6^*^/*gpg6*^ embryos exhibited a more complex, but highly consistent nodal cilia phenotype. Gross analysis of nodal cilia revealed reduced cilia length in *Atmin^gpg6/gpg6^* (Fig. 2B), a finding confirmed by systematic analysis (Fig. 2L); the modal length as well as the distribution of nodal ciliary length clearly varied, with significantly shorter cilia in mutant than in wild-type nodes. Strikingly, the number of very short (<1.5 μm) cilia present in *Atmin^gpg6/gpg6^* nodes significantly outnumbered that seen in wild type (Fig. 2L). Limb bud cilia from wild-type and mutant embryos were similarly analysed (Fig. 2D,E), and although there was a far smaller variability in these innately short cilia, a small but significant drop (*P*=0.011; Fig. 2G) in cilia length was evident in *Atmin^gpg6/gpg6^*. Similar analysis of neural tube (NT) cilia (Fig. 2H,I) again revealed a statistically significant reduction in cilia length (*P*=0.027; Fig. 2K). The nature of the *Atmin^gpg6^* mutation is such that it could conceivably encode a dominant-negative protein. No heterozygous phenotype was, however, evident in embryos or adults (data not shown) and when node cilia in heterozygous *Atmin^+/gpg6^* nodes were analysed, no obvious changes in cilia length were detected (supplementary material Fig. S4).

### Abnormal hedgehog signalling in ATMIN mutant lungs

**Fig. 3.**
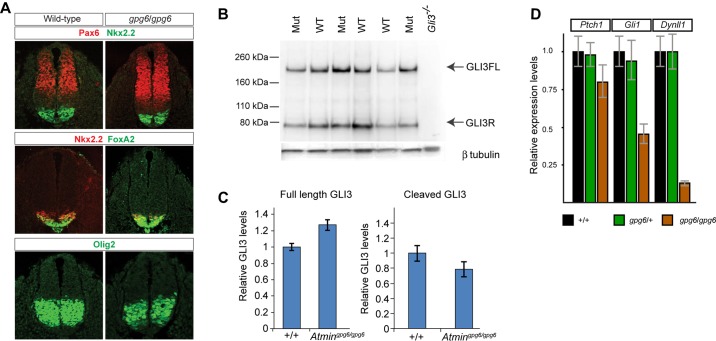
**Perturbed HH signalling in *Atmin^gpg6/gpg6^* mutant lungs.** (A) Dorsoventral patterning of 10.5 dpc *Atmin^gpg6/gpg6^* and wild-type neural tubes assessed by NKX2.2, PAX6, FOXA2 and OLIG2 immunostaining. No obvious differences are evident. (B) Western blot analysis of 9.5 dpc embryo extracts shows increased full-length 190 kDa GLI3 isoform (GLI3FL) and decreased 83 kDa cleaved repressor form (GLI3R) in *Atmin^gpg6/gpg6^* embryos when compared with wild-type controls. A *Gli3*^−/−^ embryo extract confirms the specificity of the anti-GLI3 antibody. β-tubulin was used as a loading control. (C) Quantification of the results reveals a 30% increase GLI3FL and a 20% decrease in the levels of GLI3R in the mutant embryos. (D) Hedgehog signalling was assessed by qPCR expression analysis for *Gli1* and *Ptch1*, as well as for the known target gene *Dynll1*. There is statistically significant reduction in *Gli1* expression *P*<0.01 and *Ptch1* expression *P*<0.05 (Anova).

The hedgehog (HH) signalling pathway is coordinated by primary cilia, and defective cilia are known to have an impact on HH signalling (Goetz and Anderson, 2010); previously described phenotypes include polydactyly and neural tube patterning. However, overt limb patterning was unaffected in *Atmin^gpg6/gpg6^* embryos (Fig. 1). Similarly, when dorsoventral patterning of *Atmin^gpg6^*^/*gpg6*^ and control NTs was analysed, highly similar results were obtained (Fig. 3A). Cleavage of GLI3 protein is diagnostic of HH signalling. In order to assess HH signalling in the embryo, the ratio of cleaved to uncleaved GLI3 protein was examined. In wild-type and *Atmin^gpg6^*^/*gpg6*^ embryos, both full-length and cleaved GLI3 repressor (Gli3R) were detected (Fig. 3B); small, but statistically significant variations between wild-type and mutant samples were evident, implying slightly increased HH signalling (Fig. 3C). Together, these data support the argument that although minor changes to HH signalling are present in *Atmin^gpg6/gpg6^* mutants, they are insufficient to have an impact on limb or NT patterning.

Defective HH signalling is known to affect lung patterning and can give rise to phenotypes similar to those that we describe for *Atmin^gpg6/gpg6^* (Motoyama et al., 1998). It seemed possible that defects in HH signalling were restricted to certain tissues, such as the lungs. We therefore collected embryonic lungs, but due to the small size of *Atmin^gpg6/gpg6^* lungs we were unable to readily assess GLI3 cleavage. Both *Ptch1* and *Gli1* are, however, regulated by HH and their expression provides a readout of HH signalling. When we assessed their expression by qPCR, we found *Ptch1* expression in *Atmin^gpg6/gpg6^* mutants to be ∼80% of wild-type levels and *Gli1* expression to be less than 50% of wild type (Fig. 3D). These results were consistent and statistically significant over 11 mutant lung samples, demonstrating reduced HH signalling in *Atmin^gpg6/gpg6^* lungs.

### ATMIN regulates expression of ciliogenic genes

**Fig. 4.**
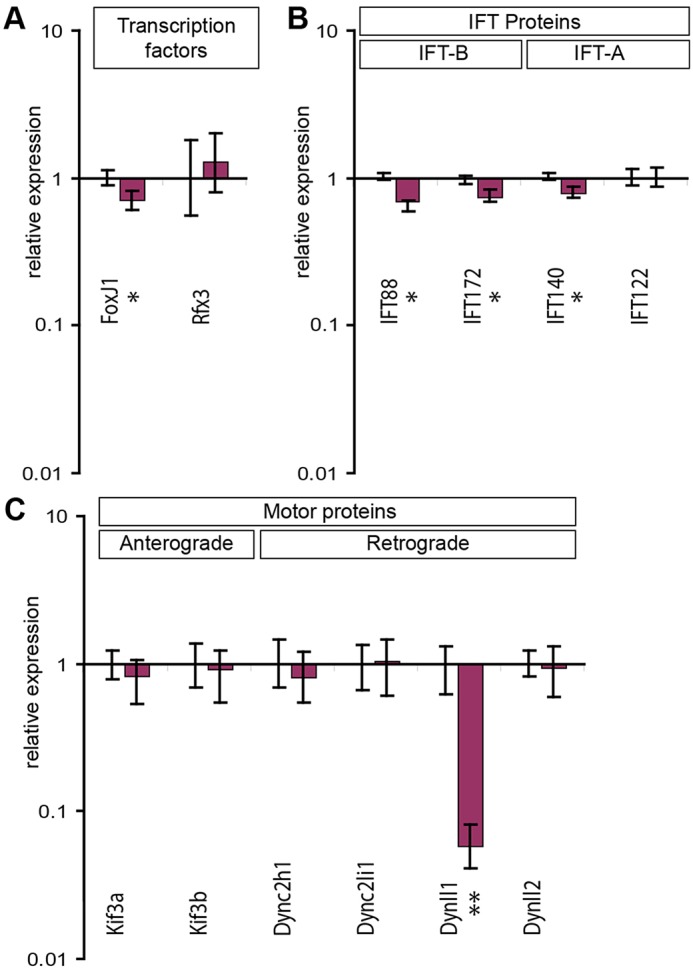
**Transcriptional profiling of ciliogenic loci reveals *Atmin* to regulate *Dynll1* expression.** Expression of ciliogenic loci was examined by qRT-PCR in 11.5 dpc *Atmin^gpg6^*^/*gpg6*^ and control embryos. (A) A mild but statistically significant (*P*<0.05) downregulation of *FoxJ1* expression is observed, whereas the expression of *Rfx3* remains unchanged (*n*=6). (B) A mild decrease in expression of anterograde IFT loci (*IFT88*, *IFT172*) and one retrograde IFT gene (*IFT140*) is observed in *Atmin^gpg6^*^/*gpg6*^ embryos. (C) *Atmin* mutation specifically downregulates transcription of *Dynll1* (*P*=1.5 e-5), but not *Dynll2* (*P*=0.26). No differences are seen in expression of the anterograde motor protein encoding loci *Kif3a* and *Kif3b* or in the retrograde motor protein encoding loci *Dync2h1* and *Dync2li1*. *P<0.05, **P<0.005.

The ATMIN protein localises to nuclei and functions as a transcriptional regulator (McNees et al., 2005; Kanu and Behrens, 2007), but it has not been reported in, and we do not detect it in, the cilium (data not shown). We therefore performed a directed expression screen to assess expression of known IFT-associated loci in 11.5 days post coitum (dpc) wild-type and *Atmin^gpg6^*^/*gpg6*^ embryos. When expression of the ciliogenic transcription factors, *Rfx3* and *FoxJ1*, was analysed, no significant difference in *Rfx3* expression was detected. However, a small, but statistically significant reduction in *FoxJ1* expression was evident in the mutant (*P*<0.05; Fig. 4A). Expression of four IFT loci was similarly analysed; two IFT-A (*Ift140*, *Ift122*) and two IFT-B (*Ift88*, *Ift172*) genes (Fig. 4B). A small but clear (∼0.3-fold) downregulation of *Ift88*, *Ift172* and *Ift140* was evident in *Atmin^gpg6/gpg6^* mutants when compared with wild-type expression. By contrast, *Ift122* expression remained equivalent between mutant and wild-type samples.

Expression of *Kif3a* and *Kif3b*, coding for subunits of the anterograde IFT motor heterotrimeric kinesin 2, showed no significant variation between wild-type and mutant samples (Fig. 4C). The known mammalian dynein 2 components *Dync2h1* and *Dync2li* were similarly examined and no statistically significant expression differences were evident between mutant and wild-type samples (Fig. 4C). The two mammalian LC8 homologues, *Dynll1* and *Dynll2*, show strong similarity at the amino acid level, differing by only six out of 89 amino acids. Although the proteins are indistinguishable by current antibodies, differences in mRNA sequence allow their expression to be differentiated. Evidence from cell lines shows that ATMIN can regulate expression of *Dynll1* (Jurado et al., 2012b); we detected a 17-fold decrease in *Dynll1* expression in *Atmin^gpg6^*^/*gpg6*^ mutant embryos compared with wild-type littermates (Fig. 4C), consistent with ATMIN similarly controlling *Dynll1* expression in the embryo. By contrast, the levels of *Dynll2* expression were equivalent between mutant and wild-type embryos (Fig. 4C).

The relationship between ATMIN and *Dynll1* expression was confirmed when ATMIN was overexpressed in mouse inner medullary collecting duct (IMCD3) cells. Transient transfection of an *Atmin*-expressing construct resulted in a 12-fold increase in the level of *Atmin* mRNA over untransfected cells (supplementary material Fig. S5). Consistent with *Atmin* regulating *Dynll1* expression, *Dynll1* mRNA levels increased by 30-fold compared with controls (supplementary material Fig. S5). Thus, *Atmin* directly or indirectly regulates *Dynll1*, but not *Dynll2*, expression in embryos.

### *Dynll1*, a potential retrograde IFT mutant recapitulating *Atmin*

Our analysis suggested a pathway whereby *Atmin* function affects expression of *Dynll1*, and to a lesser extent a number of IFT protein loci, which in turn have an impact on ciliogenesis. The strong downregulation of *Dynll1* expression suggests that loss of DYNLL1 function should recapitulate significant elements of the *Atmin^gpg6^*^/*gpg6*^ phenotype, both at the developmental and the cellular levels. In order to test this, we sourced a genetrap allele of *Dynll1* [*Dynll1^Gt(EUCE0287d04)Hmgu^*, hereafter *Dynll1^GT^*]. Analysis of mRNA levels in homozygous *Dynll1^GT/GT^* embryonic tissue demonstrated highly significant downregulation of *Dynll1* expression, consistent with it being a functional null allele (supplementary material Fig. S6). Analysis of 13.5 dpc *Dynll1^GT/GT^* embryos revealed a complex phenotype similar to that of *Atmin^gpg6/gpg6^* mutants (Fig. 1); gross oedema, exencephaly and coloboma were evident (Fig. 1M-P, Table 1). Small, mispatterned lungs were detected in almost 70% of the embryos examined (Table 1), with the remainder demonstrating no lungs. Similar to *Atmin^gpg6^*^/*gpg6*^, pulmonary isomerism was evident in a significant proportion of *Dynll1^GT/GT^* embryos (Table 1). Analysis of cardiac outflow tract development revealed high incidence of interrupted aortic arch and common outflow tract development, similar to the results seen for *Atmin^gpg6/gpg6^* mutants (supplementary material Table S1). Strikingly, additional situs defects were evident in *Dynll1^GT/GT^* embryos, with high incidence of reversed heart and stomach situs (Table 1).

**Fig. 5.**
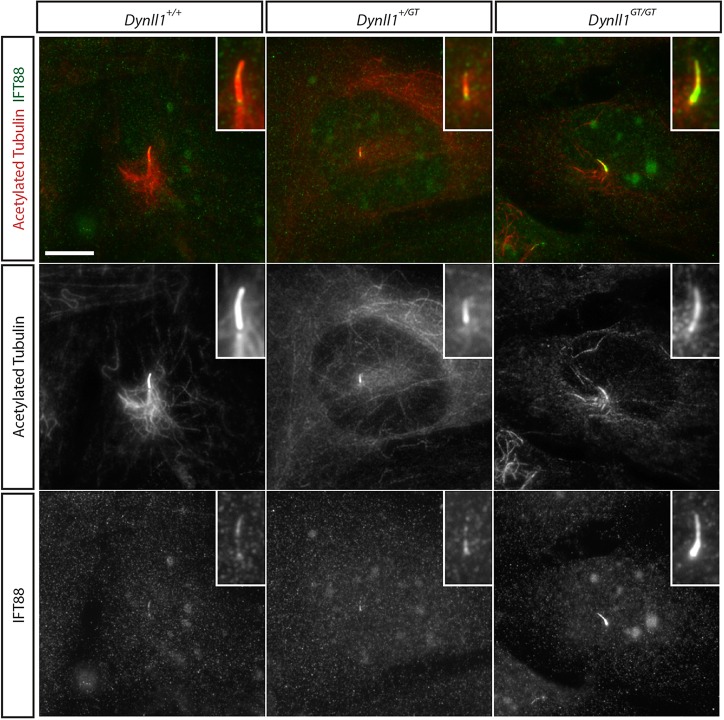
**IFT88 protein accumulates in *Dynll1^G^^T/GT^* mutant cilia.** Wild-type, *Dynll1/+* and *Dynll1/Dynll1* cells stained for the presence of IFT88 protein. Whole cells are shown in the panels with a magnified view of the cilium boxed in each panel. The bottom row shows a single channel for IFT88 in monochrome, the middle row a monochrome image for acetylated tubulin and the top row a merged image with IFT88 in green and acetylated tubulin in red. There is particular accumulation of IFT88 protein towards the base of the cilium. See supplementary material Fig. S8 for additional examples.

Analysis of nodal cilia from *Dynll1^GT/GT^* revealed these to be shorter than wild-type controls (Fig. 2C,L), although the phenotype was less extreme than in *Atmin^gpg6^*^/*gpg6*^ nodes (Fig. 2B,L). However, these cilia showed a bulging morphology, with a high incidence of bulges around the base of the cilia (Fig. 2C), a phenotype highly reminiscent of that reported for *Dync2h1* mutant embryos (Huangfu and Anderson, 2005; Ocbina et al., 2011). Examination of limb bud and neural tube cilia revealed no significant changes in cilia length, but did reveal changes in cilia morphology with bulges and/or fattening of the cilia evident (*P*=0.017; Fig. 2F,J; supplementary material Fig. S7D-H). These defects are highly characteristic of defective retrograde IFT (Satir et al., 2010). To further assess this phenotype we derived mutant (*Dynll1^GT/GT^*) and wild-type embryonic fibroblasts. We analysed IFT protein localisation by staining for the IFT protein IFT88. This revealed greatly increased staining in *Dynll1^GT/GT^* cilia compared with wild-type controls (Fig. 5), a phenotype repeated in all cilia visualised (supplementary material Fig. S8). Accumulation of the IFT protein in mutant cilia was particularly evident towards the base of the cilium (Fig. 5; supplementary material Fig. S8), consistent with the bulging seen in nodal cilia. Together, these data suggest a defect in retrograde IFT.

### DYNLL1 rescues ciliogenesis defects in cells lacking *Atmin*

**Fig. 6.**
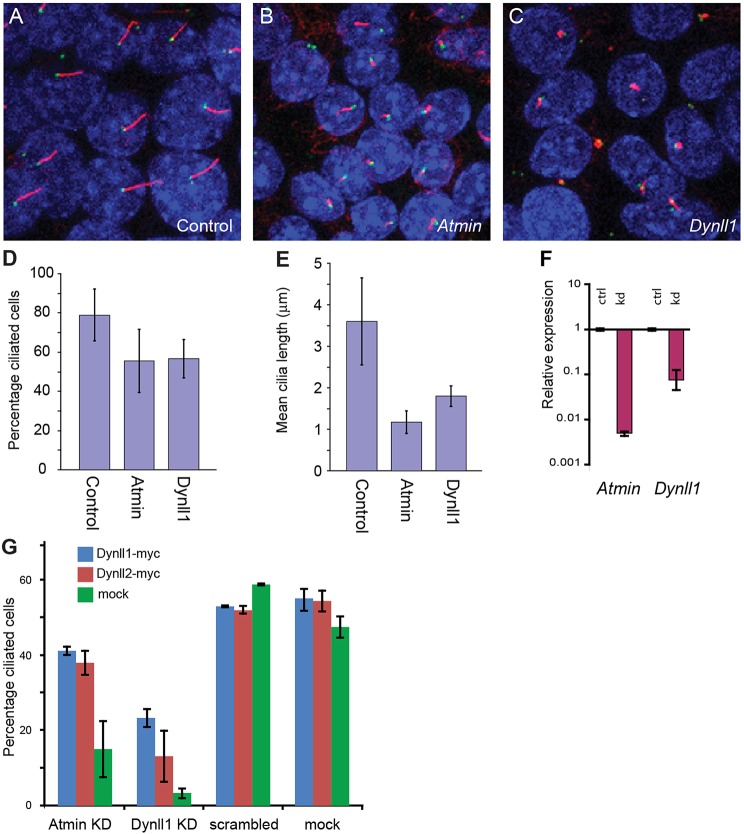
**DYNLL1 expression rescues the *in vitro* ciliogenic defects caused by loss of ATMIN.** (A) Control IMCD3 cells form normal cilia on serum starvation. (B) siRNA-mediated knockdown of *Atmin* results in reduced cilia number and reduced cilia length. Following siRNA-mediated knockdown of *Dynll1* both the frequency and the length of cilia is strongly reduced (C). Quantitation of changes in percentage ciliated cells (D) and mean cilia length (E). (F) qRT-PCR analysis showing decreased *Atmin* and *Dynll1* expression (relative to β-actin) upon *Atmin* knockdown (kd); expression is normalised to mock knockdown controls (crtl). (G) Overexpression of DYNLL1-myc and DYNLL2-myc rescue the *Atmin* and *Dynll1* knockdown ciliary phenotype compared to scrambled siRNA and mock control experiments.

In light of these results on the *Atmin* and *Dynll1* mutants, we then investigated the involvement of both proteins in mammalian ciliogenesis *in vitro*. IMCD3 cells produce clearly discernible cilia in culture (Fig. 6A); using short-interfering RNA (siRNA), we individually knocked down *Atmin* and *Dynll1*. Consistent with the analysis of *Atmin^gpg6/gpg6^* embryos, *Atmin* knockdown disrupted ciliogenesis (Fig. 6B). A similar effect was observed upon *Dynll1* knockdown (Fig. 6C). The proportion of ciliated cells dropped from 78.8% in controls, to 55.7% and 56.8% when *Atmin* and *Dynll1*, respectively, were knocked down (Fig. 6D). More strikingly, the length of the remaining cilia was reduced following knockdown, with a control length of 3.6 μm being reduced to 1.2 μm and 1.8 μm following *Atmin* and *Dynll1* knockdown, respectively (Fig. 6E). When mRNA from the siRNA-mediated knockdown of *Atmin* was analysed, both *Atmin* and *Dynll1* mRNA were found to be highly downregulated (Fig. 6F). Importantly, overexpression of either MYC-DYNLL1 or MYC-DYNLL2 rescued the *Atmin* knockdown phenotype, leading to an almost threefold increase in the number of ciliated cells in the *Atmin* knockdown (Fig. 6G). A similar fold increase was observed in *Dynll1* knockdown cells when either MYC-DYNLL1 or MYC-DYNLL2 was expressed, demonstrating that DYNLL2 can rescue at least aspects of DYNLL1 loss. Together, these data provide strong evidence for *Atmin* regulating *Dynll1* expression, which in turn regulates ciliogenesis.

### DYNLL1 localises to cilia and interacts with the putative dynein intermediate chain WDR34

**Fig. 7.**
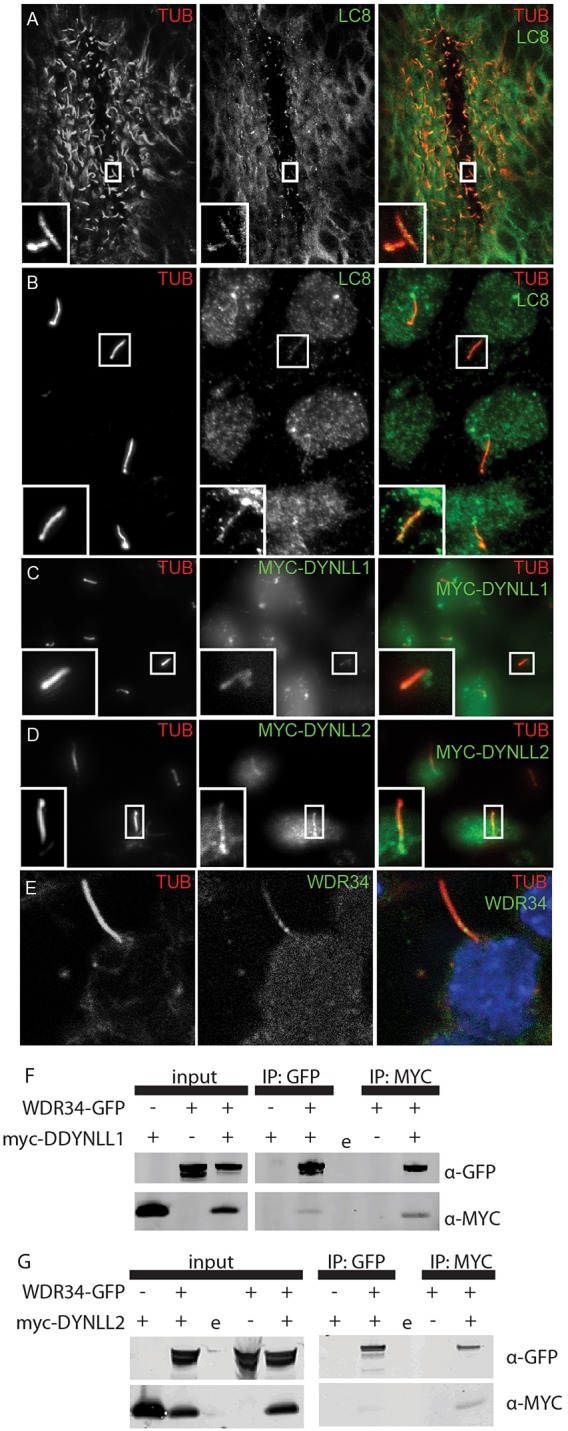
**LC8 localises to cilia and interacts with WDR34.** LC8 antibody localises to nodal cilia (A) and to primary cilia in IMCD3 cells (B). Both myc-DYNLL1 (C) and myc-DYNLL2 (D) localise to the base of the cilium, and to the ciliary axoneme. (E) GFP-WDR34 localises to the ciliary axoneme. (F) Co-expression of Wdr34-GFP and myc-Dynll1: immunoprecipitation (IP) with GFP co-precipitates a band detectable with anti-myc antibodies and IP with myc co-precipitates a band detectable with anti-GFP antibodies. (G) Co-expression of WDR34-GFP and myc-DYNLL2: IP with GFP co-precipitates a band detectable with anti-myc antibodies and IP with myc co- precipitates a band detectable with anti-GFP antibodies. ‘e’ indicates empty lane.

Although the full constitution of the mammalian cytoplasmic dynein 2 remains uncertain (Pfister et al., 2006), the *Chlamydomonas* DYNLL1/2 homologue LC8 is known to comprise part of the retrograde IFT dynein motor (Pazour et al., 1998; Rompolas et al., 2007). We therefore postulated that DYNLL1 is likely to comprise a component of mammalian cytoplasmic dynein 2. If this were the case, DYNLL1 must be present within cilia. Due to their high similarity, no isoform-specific antibodies exist for DYNLL1 and DYNLL2. We therefore stained for the presence of LC8 (DYNLL1 and DYNLL2) within nodal cilia, finding it to be present (Fig. 7A). In *Chlamydomonas* LC8 is also known to be a component of axonemal dynein (Piperno and Luck, 1979; Pfister et al., 1982; King and Patel-King, 1995) and as such might be expected to be uniformly present along the length of the motile nodal cilia. A nonmotile fraction of nodal cilia, however, lack axonemal dynein (McGrath et al., 2003). Our findings show all cilia within the node to be positive for LC8 staining (supplementary material Movie 1 and Fig. S9); moreover, the staining was punctate, suggesting localised concentrations, consistent with IFT particles.

To further confirm these data we analysed cilia in serum-starved IMCD3 cells. These immotile primary cilia lack axonemal dynein (Satir and Christensen, 2007), allowing the presence of LC8 protein within the IFT-dynein to be more easily assessed. We again detected the presence of LC8 in cilia, in addition to a strong signal at the base of cilia (Fig. 7B); puncta within the nucleus were also evident. The same result was found in NIH3T3 cells (supplementary material Fig. S10). In order to distinguish DYNLL1 and DYNLL2 we visualised MYC-DYNLL1 and MYC-DYNLL2 expressed in IMCD3 cells (Fig. 7C,D). The myc tag was detected in the nucleus and at the base of cilia, as well as within the ciliary axonemes for both DYNLL1 and DYNLL2, consistent with both proteins existing within cytoplasmic dynein 2. A highly similar result was evident when an HA-tagged DYNLL1 construct was visualised in IMCD3 cells (supplementary material Fig. S11).

*Chlamydomonas* LC8 directly interacts with the WDR34 homologue FAP133, a dynein intermediate chain within the retrograde IFT dynein 2 motor (Rompolas et al., 2007). In the absence of a WDR34-specific antibody we engineered a WDR34-GFP expression construct. When the cellular localisation of GFP-tagged WDR34 was examined in transfected, serum-starved IMCD3 cells, it was found to localise to primary cilia (Fig. 7E), consistent with it forming part of the IFT dynein. We then tested for physical interaction between DYNLL1, DYNLL2 and WDR34 by heterologous expression in human embryonic kidney (HEK293T) cells followed by immunoprecipitation. First, we confirmed that both myc-DYNLL1 and myc-DYNLL2 proteins were expressed in HEK293T cells and detectable by an anti-LC8 antibody (supplementary material Fig. S12). We then co-transfected cells with WDR34-GFP and either MYC-DYNLL1 (Fig. 7F) or MYC-DYNLL2 (Fig. 7G) and collected protein. Upon immunoprecipitation with either anti-MYC or anti-GFP antibodies a second protein positive for the other tag was detected, demonstrating co-precipitation and hence interaction of WDR34 with both DYNLL1 and DYNLL2. Together, these data strongly support the model that DYNLL1 and DYNLL2 interact with WDR34 in cytoplasmic dynein 2.

## DISCUSSION

In this study we demonstrate a novel pathway that affects ciliogenesis, comprising ATMIN and its transcriptional target *Dynll1*. The strong similarity in cilia phenotype between *Dynll1* and *Dync2h1* mutants supports the argument that loss of DYNLL1 protein has an impact on cytoplasmic dynein 2 function, an interpretation further supported by the interaction of DYNLL1 and WDR34. The documented role of DYNLL1 in mammalian cytoplasmic dynein-1 (Pfister et al., 2006) and in the *Chlamydomonas* retrograde dynein motor (Pazour et al., 1998) reinforce this model. This reveals a novel role for ATMIN, outside of the DNA damage pathway where it was first described (McNees et al., 2005), as a transcriptional regulator of both ciliogenesis and lung morphogenesis.

Two engineered null alleles of Atmin have been generated (Jurado et al., 2010; Kanu et al., 2010), and the description of their phenotypes strongly overlaps *Atmin^gpg6^*. However, all these mutants are embryonic lethal and as such postnatal roles of ATMIN, in for example DNA damage and cancer (Loizou et al., 2011), have not been easily assessed. Certain differences are evident between the reported phenotypes of the *Atmin^−^* and *Atmin^gpg^*. *Atmin^−/−^* embryos survived to a greater age than *Atmin^gpg6/gpg6^*, although these differences may relate to differing genetic background. Here we report significant cardiac outflow tract defects, a deficiency likely underlying lethality; the cardiac outflow tract is not explicitly commented on in the *Atmin^−^* analyses (Jurado et al., 2010; Kanu et al., 2010). At the cellular level, studies of *Atmin^−/−^* embryos did not report defects in ciliogenesis; however, cilia were not explicitly examined. While we see strong agreement in phenotype, we cannot rule out differences between the different alleles. The point mutations that we describe should affect DNA binding, and hence transcription factor activity, but should not have an impact on regions of the protein involved in ATM or CHK2 interaction. It is easy to imagine that phenotypes associated with ATM and CHK2 function could differ significantly between *Atmin^−^* and *Atmin^gpg6^* mutants; investigation of these potential differences await future studies.

The skeletal ciliopathies, which include SRPS, Sensenbrenner syndrome and JS, manifest as defects that include shortened limbs, thoracic constriction, retinal degeneration, cystic kidneys and congenital heart disease (Huber and Cormier-Daire, 2012). Incidence of lung mispatterning is associated with some members of the group (MIM: 263520, 208500). Strikingly, mutations in loci affecting retrograde IFT underlie a significant proportion of these disorders; mutations in the cytoplasmic dynein 2 heavy chain, *DYNC2H1*, and the IFT-A genes *IFT122*, *WDR35*/*IFT121* and *IFT43* have been reported (Huber and Cormier-Daire, 2012). Recently it has emerged that mutations in the putative cytoplasmic dynein 2 intermediate chain WDR34 underlie JS (Huber et al., 2013; Schmidts et al., 2013). Human disease mutations are often hypomorphic, making direct comparison to mouse loss-of-function alleles challenging. For example, human *DYNC2H1* point mutations lead to viable SRPS and JS individuals; in contrast, a mouse *Dync2h1* allele results in embryonic lethality and a phenotype grossly similar to *Atmin^gpg6^*, albeit with polydactyly and more severe L-R patterning defects. Similar mouse phenotypes result from mutations in the IFT-A loci *Ift122* (Cortellino et al., 2009) and *Ift121*/*Wdr35* (Mill et al., 2011); strikingly, the *Wdr35^yeti^* mutant demonstrates pulmonary hypoplasia. Other indications of the relationship between cilia and lung patterning have emerged from the study of mouse ciliopathy models (Ermakov et al., 2009; Weatherbee et al., 2009).

Pulmonary agenesis and hypoplasia are relatively rare phenotypes that have previously been reported as a result of defective HH, Wnt/beta-catenin and FGF signalling (Min et al., 1998; Motoyama et al., 1998; Li et al., 2002; Shu et al., 2002; Rajagopal et al., 2008; Goss et al., 2009). The HH pathway is known to require normal cilia to function (Goetz and Anderson, 2010), and we have demonstrated a clear impact on HH signalling in *Atmin^gpg6^* lungs; in light of these results and the obvious phenotypic overlap with the lungs of HH pathway mutants (Litingtung et al., 1998; Motoyama et al., 1998), it seems most likely that defective HH signalling underlies the pulmonary defects that we report. However, ciliopathies are by their nature pleiotropic, so it is pertinent to ask how the other pathways might be affected by cilia. A substantial literature exists on the relationship between cilia and canonical Wnt signalling (Wallingford and Mitchell, 2011); although controversy remains on this subject, it is clear that some ciliopathies do have an impact on the canonical Wnt pathway (Wallingford and Mitchell, 2011). FGF receptors have been reported to localise to cilia in the mouse node (Tanaka et al., 2005), raising a possible role for cilia in FGF signalling. FGF signalling is known to affect cilia length control (Neugebauer et al., 2009); however, the lack of other overt FGF phenotypes and the impact of *Dynll1^GT^* on ciliogenesis supports the argument against this being the cause of cilia phenotypes in *Atmin^gpg6^*.

The striking similarity in phenotype between *Atmin* and *Dynll1* mutants suggests that much of the *Atmin^gpg6^* phenotype manifests through regulation of *Dynll1* expression. However, differences exist between the two mutants. In terms of our analysis, the most striking difference is in cilia morphology. There is strong downregulation of *Dynll1* expression in *Atmin^gpg6/gpg6^* embryos, but not total loss of expression. The remaining low levels of *Dynll1* may permit sufficient retrograde IFT to prevent the basal bulging of cilia. This alone might explain why *Dynll1^GT/GT^* mutants do not phenocopy *Atmin^gpg6/gpg6^* mutants. A second possibility exists in that ATMIN regulates not only *Dynll1* expression, but also expression of other loci; we have demonstrated downregulation of *Ift88*, *Ift172*, *Ift140* and *FoxJ1*. A recent study revealed that loss of one copy of either an IFT-A (*Ift122*) or IFT-B (*Ift172*) locus partially rescues the *Dync2h1* phenotype, leading to loss of similar bulges from cilia (Ocbina et al., 2011). In the case of *Atmin^gpg6^* at least three IFT loci are downregulated, including *Ift172*. In light of the similarity of phenotype between *Dynll1 and Dync2h1* we cannot rule out a genetic interaction between targets of ATMIN; downregulation of *Dynll1* expression being partially rescued by downregulation of IFT loci.

One of the most striking results of this study is the presence of bulges at the base of *Dynll1^GT/GT^* cilia. Conventional understanding of IFT suggests that failure of retrograde transport should result in bulges at the tips of cilia, a phenotype reported for certain IFT-A mutants (Tran et al., 2008; Ocbina et al., 2011). It would be tempting to postulate that the basal bulges in *Dynll1^GT/GT^* embryos reflect functions of DYNLL1 outside of cytoplasmic dynein 2, were it not for the published data on *Dync2h1* (Ocbina et al., 2011). Both *Dynll1^GT/GT^* and *Dync2h1^lln/Iln^* embryos show bulges at the base of cilia, suggesting that this phenotype can represent defective cytoplasmic dynein 2 function. This could reflect partial loss of cytoplasmic dynein 2 function, caused by hypomorphic alleles, and/or partial functional redundancy; our data suggest that DYNLL2 may be able to function in place of DYNLL1. These data do, however, suggest a role for cytoplasmic dynein 2 in the efficient exit of IFT particles from the base of the cilium. The apparent differences between IFT-A and cytoplasmic dynein 2 mutant cilia also make it unclear whether cytoplasmic dynein 2 is the only retrograde motor functioning in the cilium. Further genetic analysis of definitive null alleles of these and additional loci may cast light on this. Unfortunately, the published analysis of *Dync2li1* mutants, the other characterised cytoplasmic dynein 2 component, provides insufficient resolution to fully assess ciliary morphology (Rana et al., 2004).

Our analysis reveals a dual role for ATMIN in both ciliogenesis and DNA damage pathways. Indeed, ciliopathy causing mutations in genes encoding DNA damage proteins, have previously been described (Chaki et al., 2012; Zhou et al., 2012). The existence of such links is not entirely unexpected, as cilia must be dismantled, releasing the centrioles before cells divide (Satir and Christensen, 2007); phosphorylation of the dynein light chain TCTEX1 is pivotal in both ciliary disassembly and cell cycle progression in ciliated cells (Li et al., 2011). Following DNA damage, cell cycle checkpoints function to prevent cell division and it seems possible that a similar function is being mediated through preventing (or simply slowing) cilia from being dismantled. The complete absence of both cilia and centrioles, however, does not affect normal DNA damage responses (Bazzi and Anderson, 2014). A contrasting hypothesis is that such interactions might sustain cilia in DNA damaged cells, maintaining the ability of such cells to receive cilia-dependent signals. Whether such interactions interlink ciliogenesis and the cell cycle remains to be determined.

In summary, we present data demonstrating a role for ATMIN transcriptional regulation of *Dynll1* in ciliogenesis. Our data further provide evidence for DYNLL1 and WDR34 comprising elements of the mammalian cytoplasmic dynein 2, taking the number of identified components from two to four. The rescue of ATMIN ciliogenesis defects by DYNLL1 reflects the major role played by this transcriptional relationship. Evidently both loci have additional well-defined roles. The variations in phenotype between the *Atmin* and *Dynll1* mutants demonstrate that transcriptional regulation of ciliogenesis is not necessarily a simple linear process.

## MATERIALS AND METHODS

### Mice

The ENU-derived *Atmin^gpg6^* mutant is as previously described (Ermakov et al., 2009). SNP-based haplotypic mapping defined a minimal region between rs13480001 and rs13480012 and candidate sequencing identified the mutation. *Atmin^H210Q^* was isolated from the Harwell ENU archive (Quwailid et al., 2004). Both strains were maintained by backcrossing to C3H/HeH. All mice were housed in IVCs in the Mary Lyon Centre. *Dynll1^Gt(EUCE0287d04)Hmgu^* ES cells were ordered from repository: chimeras were created and mice bred onto the C3H/HeH background; analysis was of incipient congenic mice. All animal work was conducted in accordance with UK law, under the auspices of Home Office licences and following local ethical approval.

### DNA constructs

Mouse *Dynll1*, *Dynll2* and human *WDR34* cDNA were PCR amplified, sequenced and shown to agree with NCBI reference sequences. cDNAs were cloned in-frame into pCMV-myc (Clontech), pCI-HA tag vector (Promega) and pEGFP-N1 (Clontech). The full-length *Atmin* cDNA from IMAGE clone 6847850 was subcloned into pCMV-Myc-N (Clontech).

### DNA damage marker analysis

Wild-type and *gpg6/gpg6* embryos, 12.5 dpc, were collected. For positive control, wild-type embryos were placed in 0.025% methyl methanesulfonate (Sigma, 129925) in DMEM medium (Gibco) for 3 h. Embryos were fixed in 4% PFA, ethanol dehydrated and paraffin embedded. 5 µm sections were taken, deparaffinised, rehydrated and subjected to heat-induced antigen retrieval at 110°C for 2 min in buffer containing 10 mM sodium citrate, 0.05% Tween-20, pH 6.0. The staining was carried out using EnVision G2 Doublestain System (Dako). The 53BP1 antibody (cat. A300-272A, Bethyl Laboratories) was used at a dilution of 1:5000 for 1 h at room temperature. For colour development, 3,3′-diaminobenzidine tetrahydrochloride (DAB^+^) and Hematoxylin were used. The slides were scanned using Aperio ScanScope scanner and analysed using ImageScope software (both Aperio Technologies).

### Protein preparation, western blotting and immunoprecipitation

Epitope-tagged constructs were transfected into HEK-293T cells using JetPei (PolyPlus Transfection) according to the manufacturer's instructions. Immunoprecipitations (IPs) and western blots were performed as previously described (Field et al., 2011). In brief, HEK-293T cells were lysed in RIPA buffer supplemented with protease inhibitor cocktail (Roche) and prepared protein was quantified using Bradford reagent (Sigma). Protein was resolved with 4-12% Bis-Tris gels (Life Technologies), transferred onto nitrocellulose membranes (iBLOT; Life Technologies), then blocked with 5% milk in PBT (0.1% Tween-20 in PBS). Membranes were probed with anti-DLC8 (1:10,000; Abcam, Ab51603), anti-GFP (1:4000; Roche, 11,814,460,001), anti-Myc (1:1000; Sigma, C3956) anti-ATMIN (Millipore, ab3271) or anti-Gli3 [1:500 (Wen et al., 2010)] primary antibodies, fluorescently conjugated rabbit and mouse secondary antibodies (1:15,000; LI-COR Biosciences), then visualised with the Odyssey imaging system (LI-COR Biosciences). IPs were performed with 0.4 mg pre-cleared lysate per IP, using 1 µg of anti-GFP (Roche, 11,814,460,001) or anti-Myc (Sigma, C3956) antibodies bound to Protein G Sepharose beads (Sigma).

### Cell culture, transfection and immunofluorescence

IMCD3 cells were grown in DMEM/F12 (Gibco) media supplemented with 5% foetal bovine serum (Life Technologies), and pen-strep (Life Technologies) on 13 mm glass coverslips, thickness No. 0. NIH3T3 were similarly cultured but in DMEM (Gibco) media supplemented with 10% foetal bovine serum (Life Technologies). Constructs were transfected using JetPei (Polypus Transfection) according to the manufacturer's instructions. Between 6 and 18 h post transfection, growth medium was changed to 0% serum medium and cells were left ‘serum starved’ for 72 h, to encourage ciliation.

Cells were pre-extracted for 30 s in 0.5% Triton X-100 in PHEM buffer (Schliwa and van Blerkom, 1981), then fixed in 4% PFA in PHEM buffer at 37°C for 15 min. Embryos dissected in cold PBS were fixed in 4% PFA in PBS for 20 min at room temperature, then washed in 0.5% SDS in PBS three times, 10 min per wash. Staining for Myc and HA protein tags was with goat anti-Myc (1:200; Abcam, ab9132) and mouse anti-HA (1:100; Covance, MMS-191R). Cilia were labelled using anti-detyrosinated tubulin (Glu-tubulin, 1:200; Millipore, AB3201). Endogenous LC8 was detected using rabbit anti-LC8 (1:500; Abcam, ab51603) and cilia with an antibody against acetylated tubulin (1:500; Sigma, T7451). IFT88 (1:500; Abcam, ab42497). Visualisation utilised Dylight 488 mm and 650 mm secondary antibodies (1:250; Abcam, ab9635, ab96875, ab96894). Slides were visualised on an Axio Observer Z1 inverted microscope (Zeiss) fitted with an Apotome attachment, or a LSM700 confocal. For neural tube markers, Nkx2.2 (mouse; DSHB, 74.5A5), Olig2 (rabbit; Millipore, AB9610), Pax6 (rabbit; Millipore, AB2237), FoxA2 (goat; Santa Cruz, sc-6554X) were used.

### siRNA-mediated knockdown

For RNAi knockdown siRNAs were transfected into ∼60% confluent IMCD3 cells utilising Lipofectamine RNAiMax (Life Technologies), as previously described (Dawe et al., 2009). Post transfection, cells were serum starved for 96 h before staining and imaging. Knockdown was confirmed by qRT-PCR. Transfection efficiency was assessed using Block-iT Alexa Fluor Red Fluorescent Control (Invitrogen), confirming high level transfection. Pools of three siRNAs were used for *Atmin*; MSS214922, MSS214923 and MSS214924 (Invitrogen) and for *Dynll1*; S80610, S80611, S80612 (Invitrogen). Scrambled control was ON-TARGET plus Non-Targeting Pool, D-001810-10 (Thermo Scientific). Cilia number and length were measured manually from ten fields of view of ×100 magnified images and statistical significance of differences between scrambled and *Dynll1* and *Atmin* knockdowns was assessed using Student's *t*-tests.

### Quantitative PCR

One mg RNA was isolated from wild-type and *Atmin^gpg6/gpg6^* E11.5 littermates using the RNeasy Mini Kit (Qiagen) and cDNA was prepared for qPCR using the High Capacity cDNA Reverse Transcription Kit (ABI). Quantitative real-time PCR was performed in triplicate on six different embryos of each genotype for the genes *FoxJ1*, *Rfx3*, *Ift88*, *Ift172*, *Ift140*, *Ift122*, *Kif3a*, *Kif3b*, *Dync2h1*, *Dync2li1*, *Dynll1*, *Dynll2* and *Atmin*. For *Gli3* and *Ptch1* analysis, 11 *Atmin^gpg6/gpg6^*, 8 *Atmin^gpg6/+^* and 10 *Atmin*^+/+^ lungs were analysed. Endogenous control was beta actin (*Actb*). All assays were provided by ABI. Alterations in gene expression in *Atmin^gpg6/gpg6^* were expressed relative to the mean intensity in wild-type embryos, which was given a standardised value of 1. Negative controls of reactions without cDNA template were included.

### Electron microscopy

For morphological analysis of cilia, embryos, limb buds and neural tube portions were fixed in 2.5% glutaraldehyde and 1% osmium tetroxide, dehydrated through an ethanol series, transferred into acetone, critical point dried (EMITECH 850), mounted, sputter coated with platinum (8 nm; QUORUM 150RS) and viewed in a JEOL 6010LV scanning electron microscope. Tissue was collected in parallel for genotyping. For nodes four wild-type and four mutant nodes were analysed, all visible cilia measured and plotted for frequency against length. For neural tubes four wild-type and four mutant samples were analysed; ∼140 cilia were measured per sample. For limb buds three wild-type and three mutant samples were compared; between 50 and 150 cilia were measured per sample. Two-tailed *t*-test was used to test for statistical significance of differences.

## Supplementary Material

Supplementary Material
