# Supplementary material for: ATMIN is a transcriptional regulator of both lung morphogenesis and ciliogenesis
Source: Development. 2014 Oct;141(20):3966–77. doi: 10.1242/dev.107755 (PMC4197704; doi:10.1242/dev.107755)
Supplement: Supplementary Material [file supp_141_20_3966__index.html]

Supplementary Material 

# ATMIN is a transcriptional regulator of both lung morphogenesis and ciliogenesis

## DEV107755 Supplementary Material

**Files in this Data Supplement:**

- **Supplementary Material**
