# Supplementary material for: ATMIN is a transcriptional regulator of both lung morphogenesis and ciliogenesis
Source: Development. 2014 Oct;141(20):3966–77. doi: 10.1242/dev.107755 (PMC4197704; doi:10.1242/dev.107755)
Supplement: Supplementary Material [file supp_141.20.3966_DEV107755.pdf]

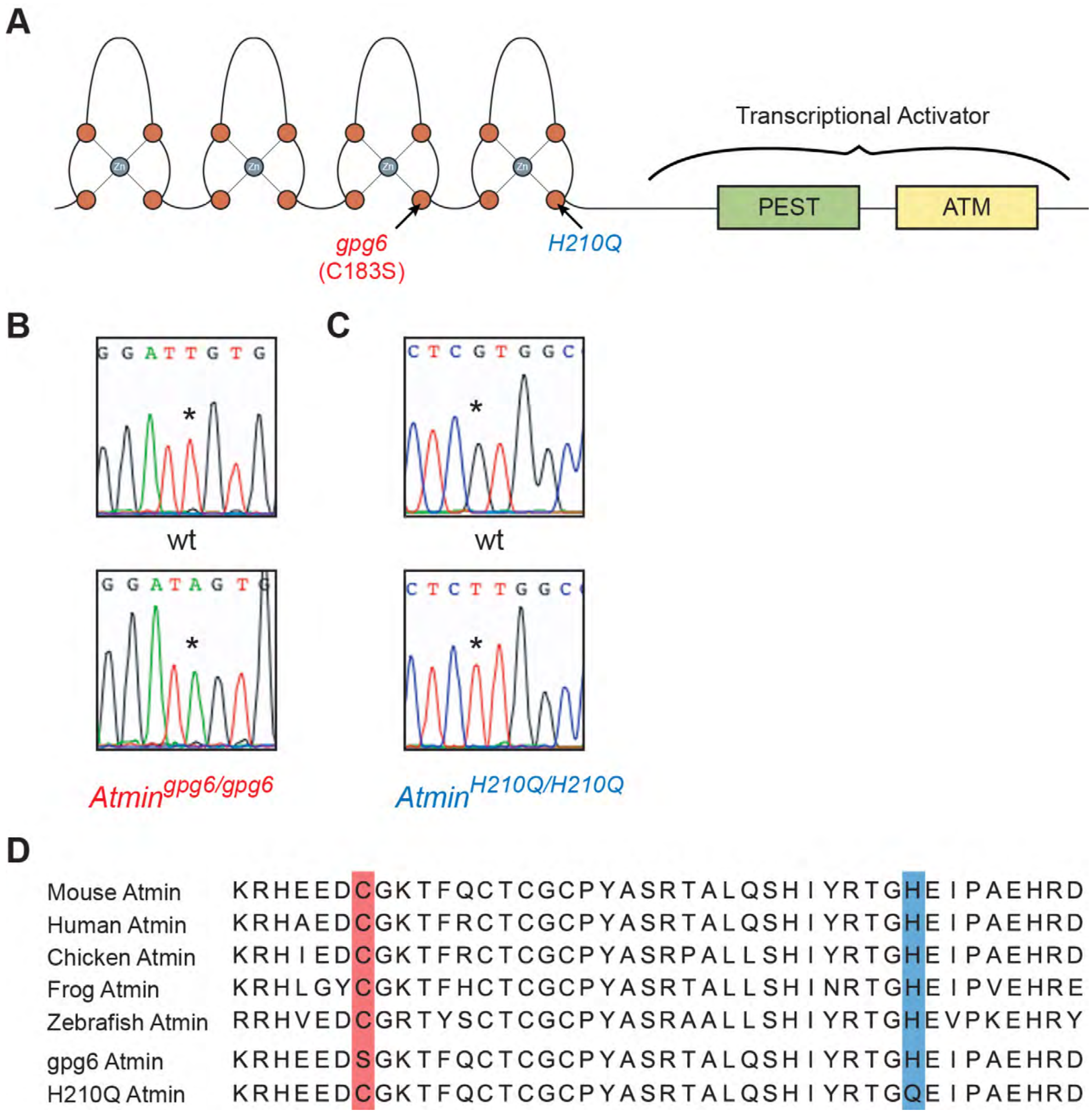

Supp. Figure 1. Zinc finger mutations in *Atmin*. (A) Diagrammatic representation of Atmin (not to scale) showing 4 zinc fingers, a PEST domain, an ATM binding domain and the region associated with transcriptional activation. The positions of the two mutations are indicated. Sequence traces showing the *gpg6* mutation (B) and the *H201Q* mutation (C). (D) Multi-species alignment of ATMIN reveals the mutated amino acids to be highly conserved.

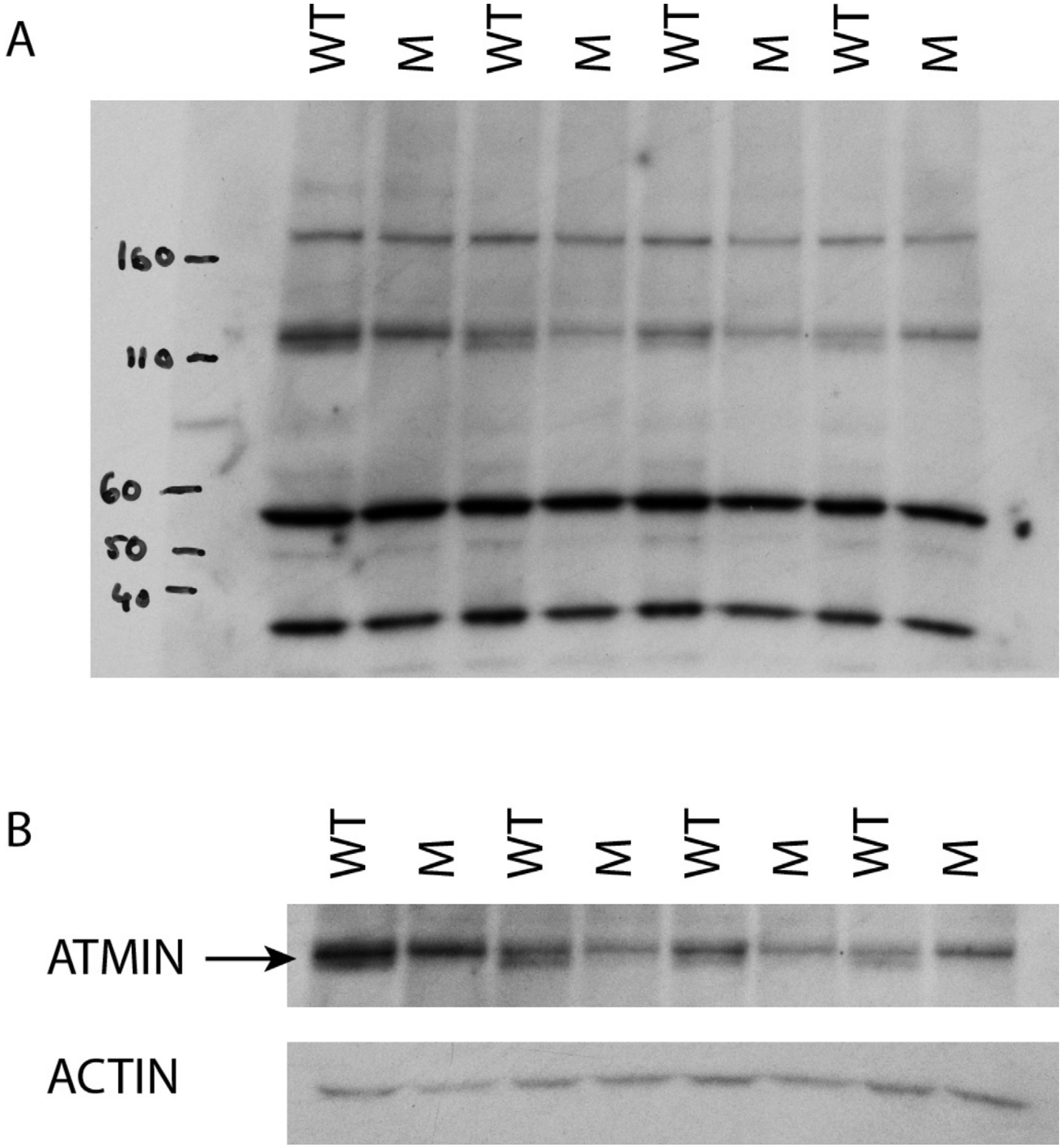

Supp. Figure 2. ATMIN antibody detects multiple bands. (A) Western blot analysis of 11.5dpc embryo extracts shows the antibody to detect multiple band including a pair at ~110-120 kDA. (B) The lower of the two ATMIN bands is undetectable in gpg6 homozygotes. The Actin loading control is shown below.

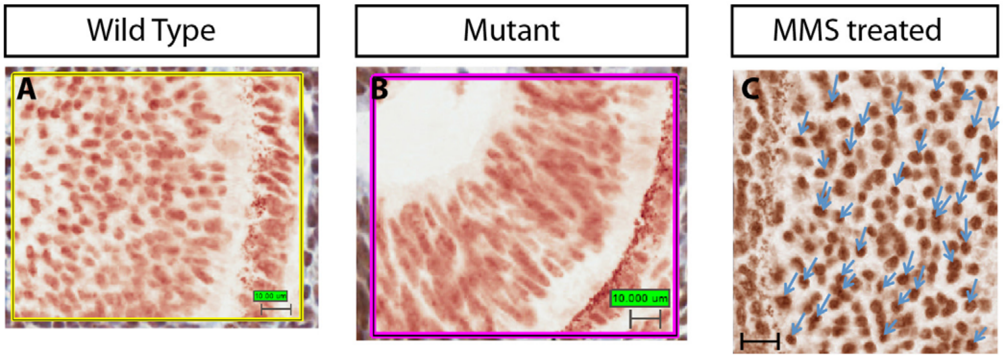

D

Genotype/parameter	wild type	*gpg6/gpg6*
Average positive intensity	171.531	163.738
Cells with medium positive signal, %	48.28	49.15
Cells with strong positive signal, %	2.09	2.10

12.5 dpc wild type and gpg6/gpg6 embryos were collected. For postive control, wild type embryos were placed in 0.025% methyl methanesulfonate (Sigma, 129925) in DMEM medium (Gibco) for 3 hours. Embryos were fixed in 4% PFA, ethanol dehydrated and paraffin embedded. 5 µm sections were taken, deparaffinised, rehydrated and subjected to heat-induced antigen retrieval at 110°C for 2 min in buffer containing 10 mM sodium citrate, 0.05% Tween-20, pH 6.0. The staining was carried out using EnVision G2 Doublestain System (Dako, Denmark). The 53BP1 antibody (cat. A300-272A, Bethyl Laboratories) was used at a dilution of 1:5000 for 1 h at room temperature. For color development, 3,3'-diaminobenzidine tetrahydrochloride (DAB+) and hematoxylin were used. The slides were scanned using Aperio ScanScope scanner and analysed using ImageScope software (both Aperio Technologies, United States).

Supp. Figure 3. DNA damage. Wildtype (A), *Atmin*$^{gpg6/gpg6}$ (B) and wild type MMS treated (C, positive control) 12.5 dpc embryos were stained for 53BP1 localisation. Sections of the eye are shown. (D) Analysis of staining patterns between wildtype and *Atmin*$^{gpg6/gpg6}$ embryos reveals no significant differences.

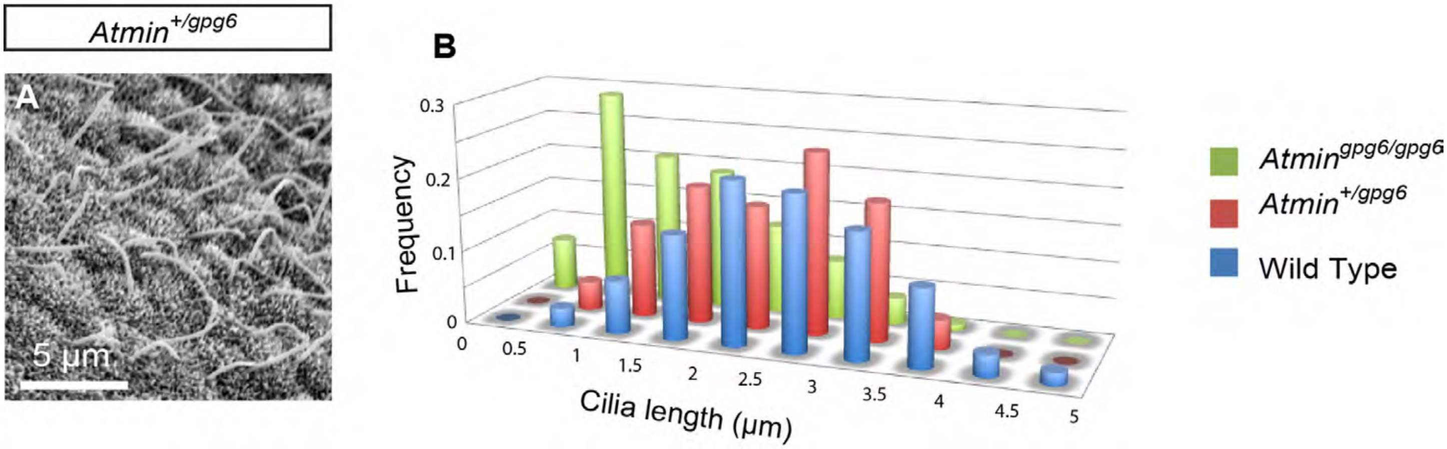

Supp. Figure 4. No cilia phenotype evident in *gpg6* heterozygotes. (A) SEM image of *gpg6/+* node, showing a similar phenotype to wild type embryos. (B) categorical analysis of cilia length from *gpg6/+* embryos compared to wild type and *gpg6/gpg6* embryos. No significant difference is evident between the wild type and *gpg6/+* embryos.

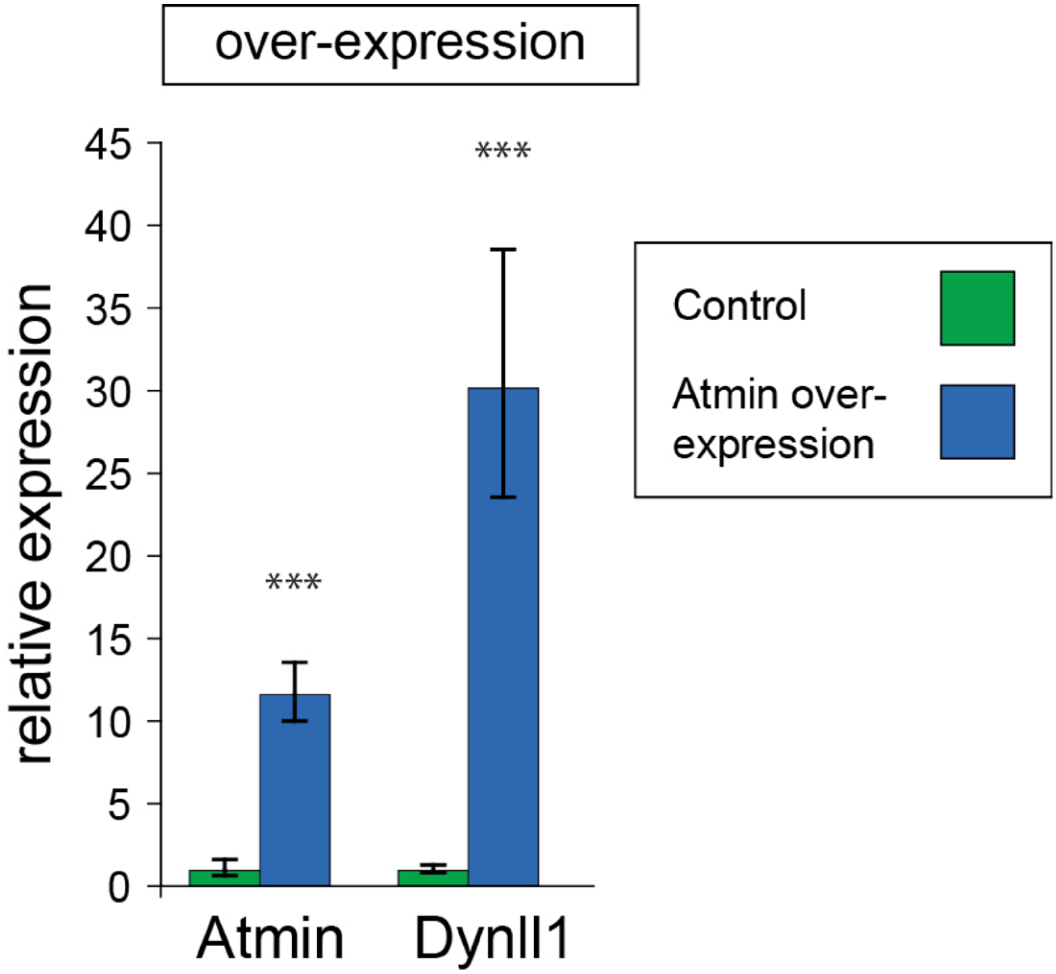

Supp. Figure 5. Overexpression of *Atmin* in IMCD3 cells results in increased *Dynll1* expression. n=3 repeats. qRT-PCR analysis revealed a 30-fold increase in *Dynll1* expression when *Atmin* expression was induced by 10-fold. *** represents p<0.001 and error bars show standard deviation.

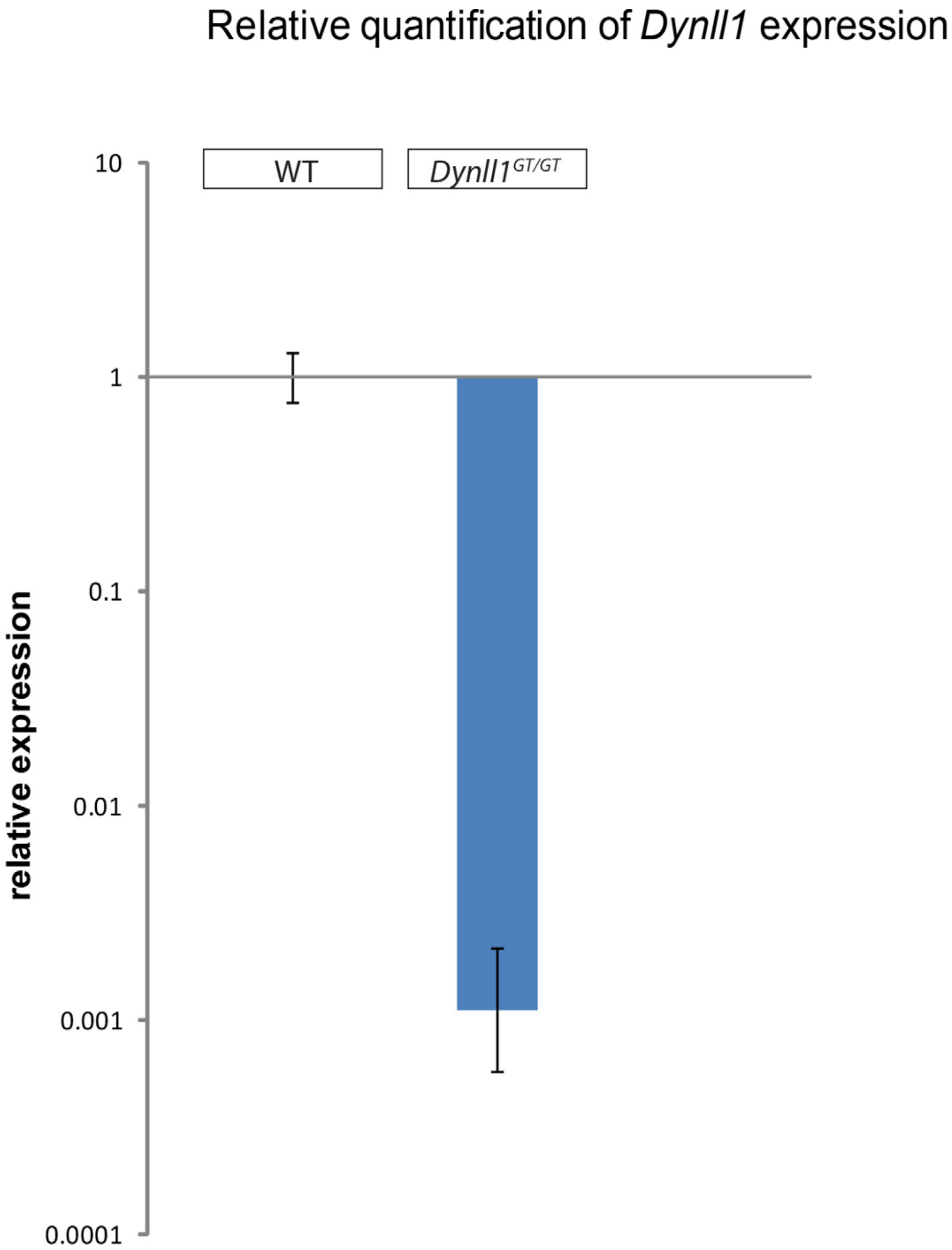

Supp. Figure 6. No *Dynll1* expression was detected in *Dynll1*$^{GT/GT}$ mutant. qRT-PCR analysis of *Dynll1* expression in wild type (WT) and *Dynll1*$^{GT/GT}$ embryonic liver.(n=6).

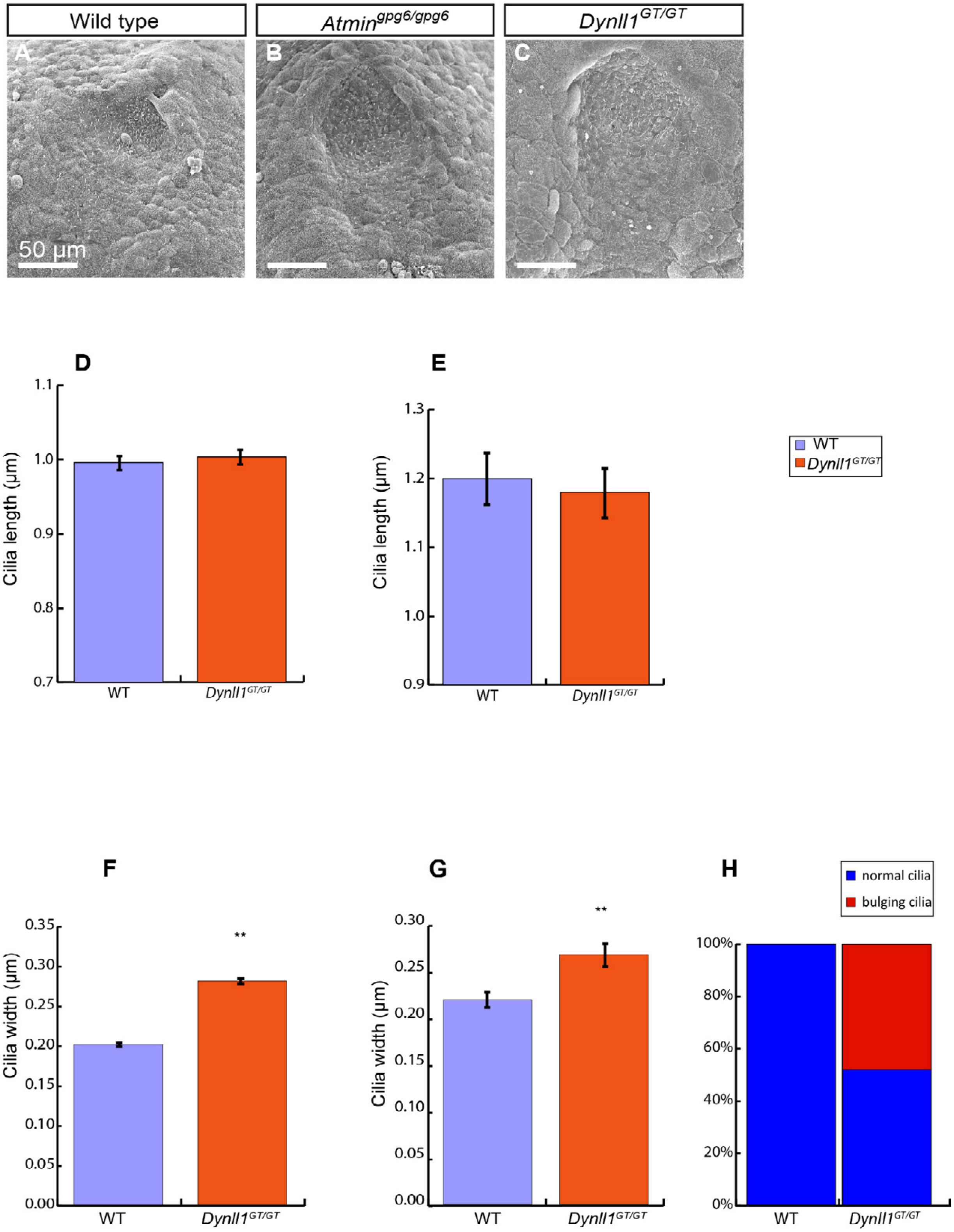

Supp. Figure 7. *Dynll1$^{GT/GT}$* mutant cilia demonstrate significant incidence of morphological abnormalities. SEM analysis of nodes from 2-4 somite stage wild type (A), *Atmin$^{gpg6/gpg6}$* (B) and *Dynll1$^{GT/GT}$* (C) embryos, revealing no obvious gross morphological differences at this magnification. Analysis of neural tube (D) and limb bud (E) cilia length from 11.5 dpc wild type (WT) and *Dynll$^{GT/GT}$* embryos did not reveal statistically significant changes (p=0.183 and p=0.425 respectively). Three embryos were analysed per class. Analysis of cilia width (measured 0.5㎜m above the cell membrane) from the same samples in neural tube (F) and limb bud (G) cilia however, revealed significantly wider cilia in mutants (p=0.0166 and p= 0.173 respectively). (H) Categorical analysis of proportion of cilia falling into either "normal" or "bulging" classes for WT or mutant *Dynll$^{GT/GT}$* nodes. Approximately half of the cilia in the mutant nodes presented the bulging phenotype.

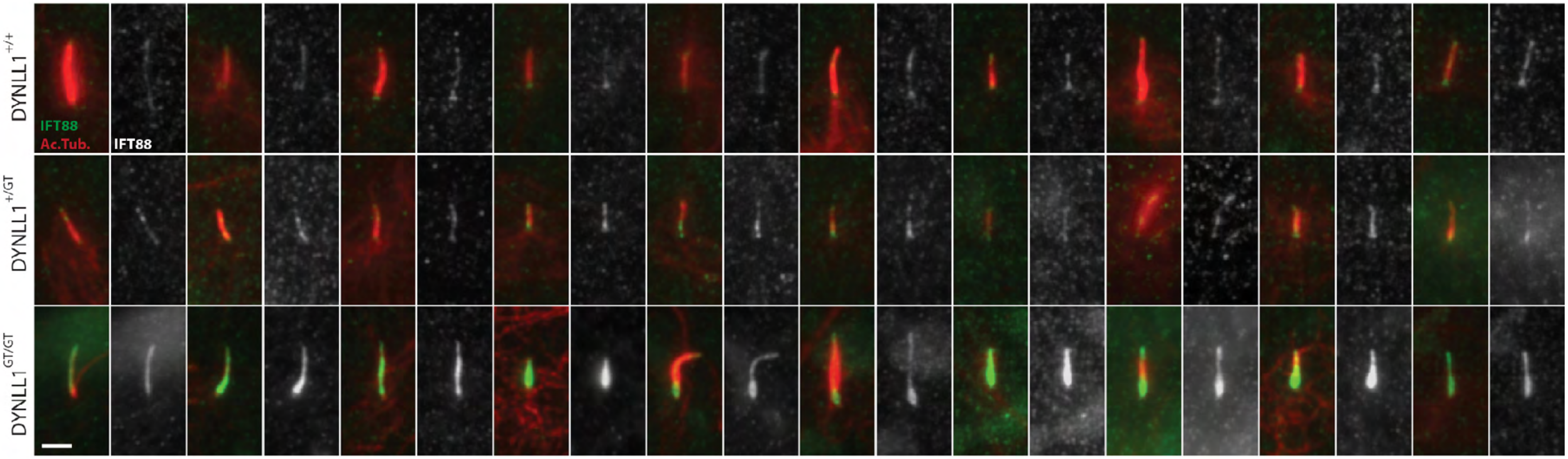

Supp. Figure 8. IFT88 protein accumulates in *Dynll$^{GT/GT}$* mutant cilia. Wild type, *Dynll1/+* and *Dynll1/Dynll1* cilia stained for the presence of IFT88 protein (green) and acetylated tubulin (red). Ten randomly selected cilia were imaged for each genotype, using identical imaging conditions and exposure. The IFT88 channel is shown in monochrome to the right of each merged image.

LC8 in node

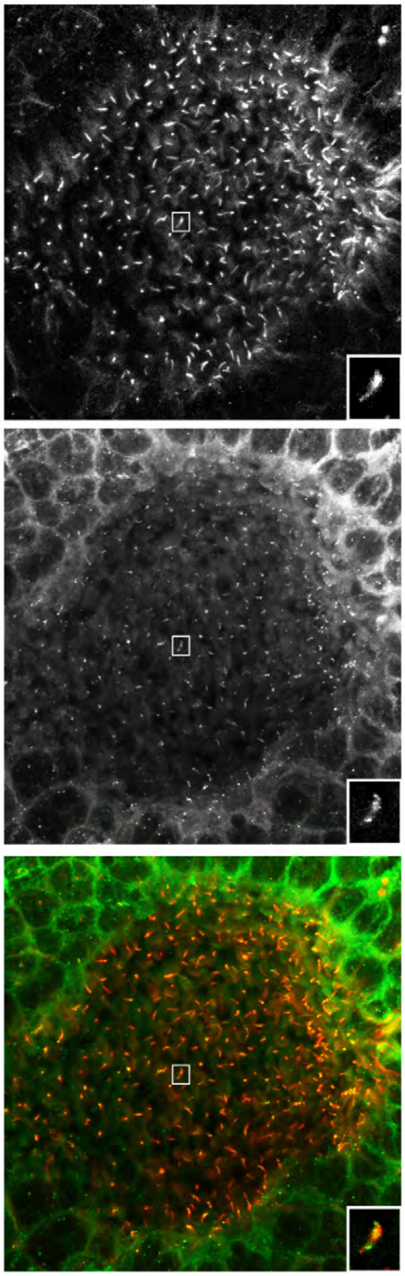

Supp. Figure 9. LC8 localises to nodal cilia. A maximum intensity projection of the node imaged in Supp. Movie. Top panel: anti-acetylated tubulin antibody, visualising cilia. Middle panel, anti LC8 antibody. Bottom panel shows combined image with acetylated tubulin in red and LC8 in green. A representative cilium (boxed) is shown magnified in the bottom right hand corner of each panel.

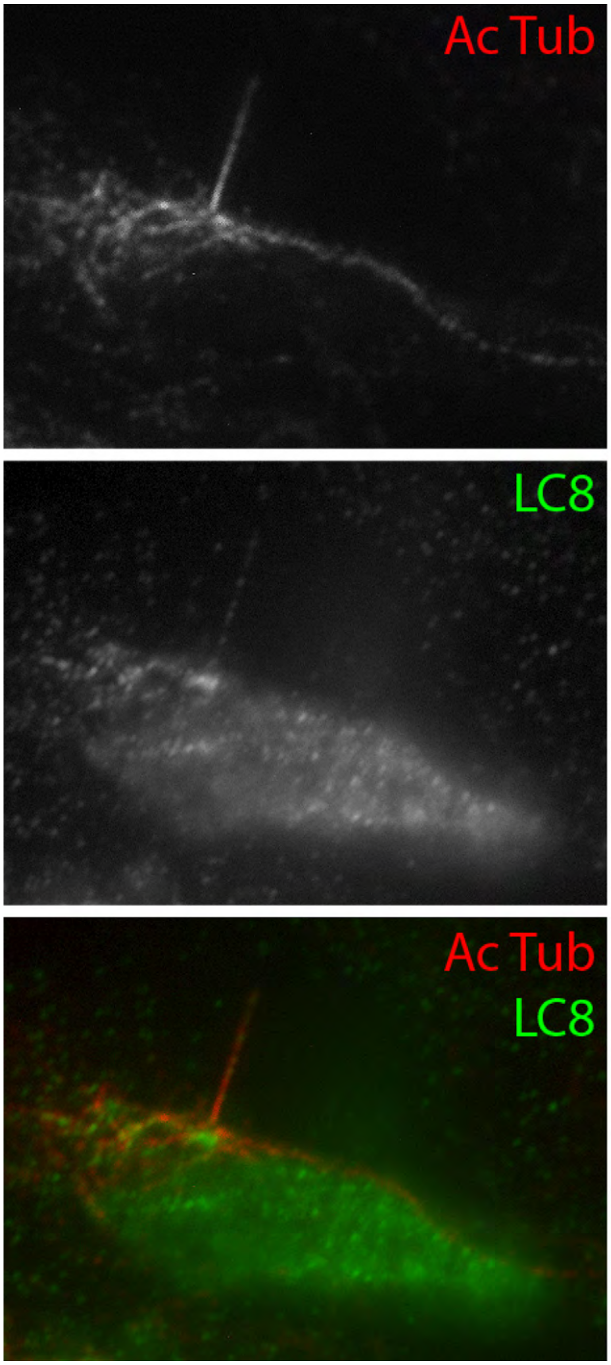

Supp. Figure 10. LC8 localises to primary cilia in NIH-3T3 cells. A cultured NIH-3T3 cell labelled for acetylated tubulin (top panel) and for LC8 (middle panel) revealing punctate LC8 staining in the cilium. A combined image with acetylated tubulin in red and LC8 in green is shown in the bottom panel.

Ciliated IMCD3 cells transfected with HA-Dynll1

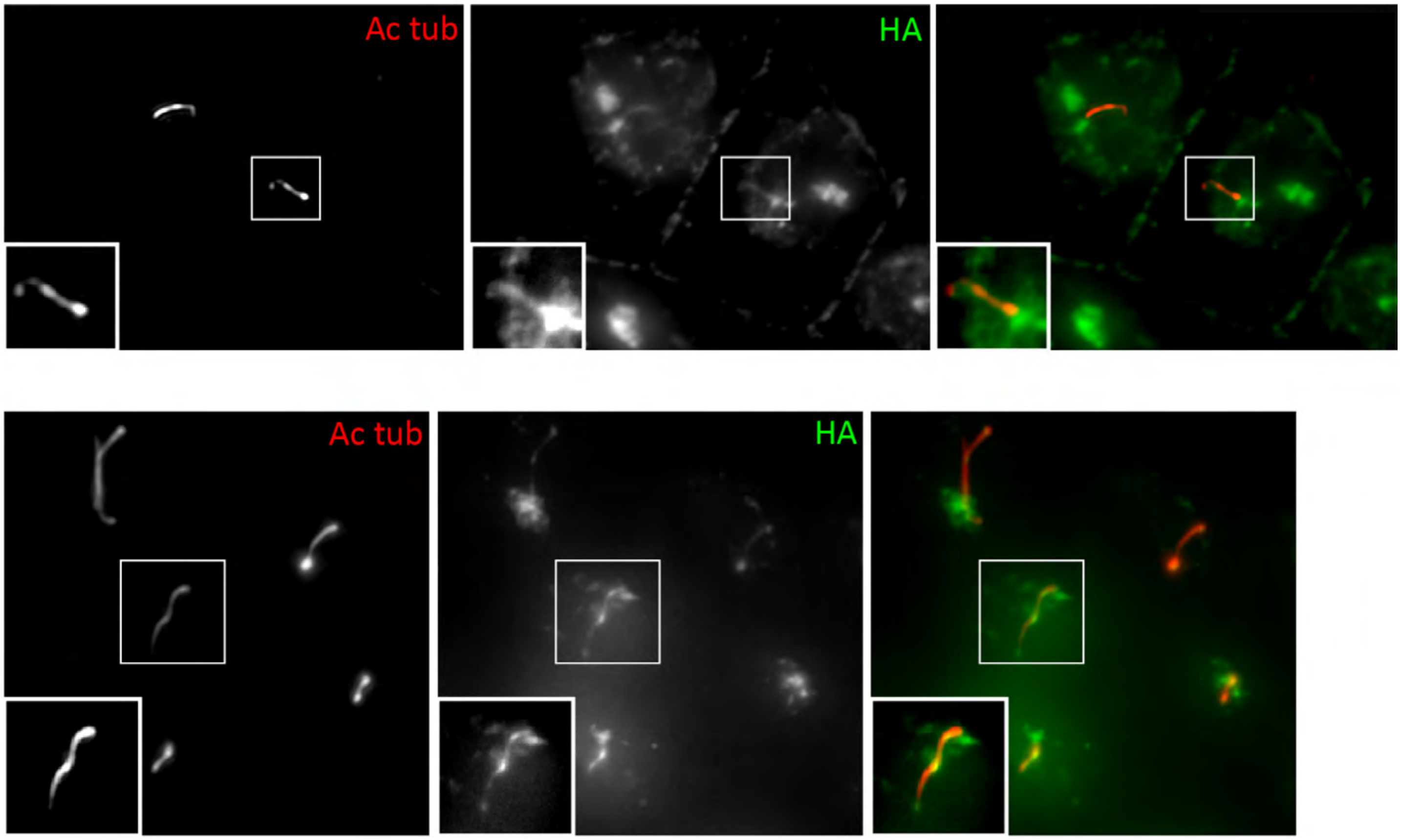

Supp. Figure 11. HA-DYNLL1 localises to primary cilia in cells. Cultured ciliated IMCD3 cells expressing HA-DYNLL1 visualised with anti-HA (green) and anti-acetylated tubulin (red), revealing localisation of DYNLL1 to the cilium.

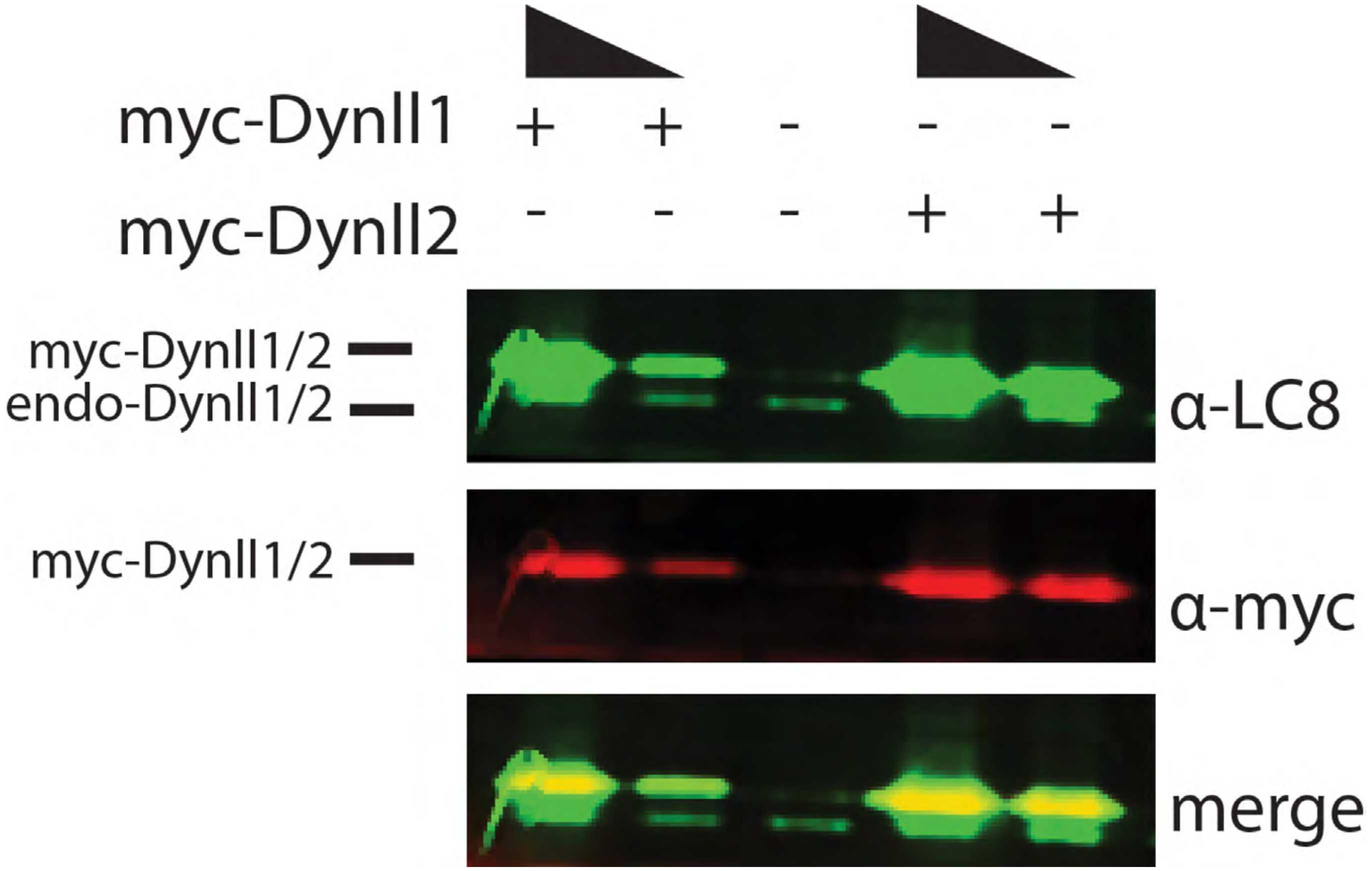

Supp. Figure 12. LC8 expression in HEK293T cells. Myc-DYNLL1 and myc-DYNLL2 expressed in HEK293T cells, revealing endogenous protein at ~10kDa detected by anti-LC8 antibody and larger myc-tagged proteins identified by both anti-myc and anti-LC8 antibodies.

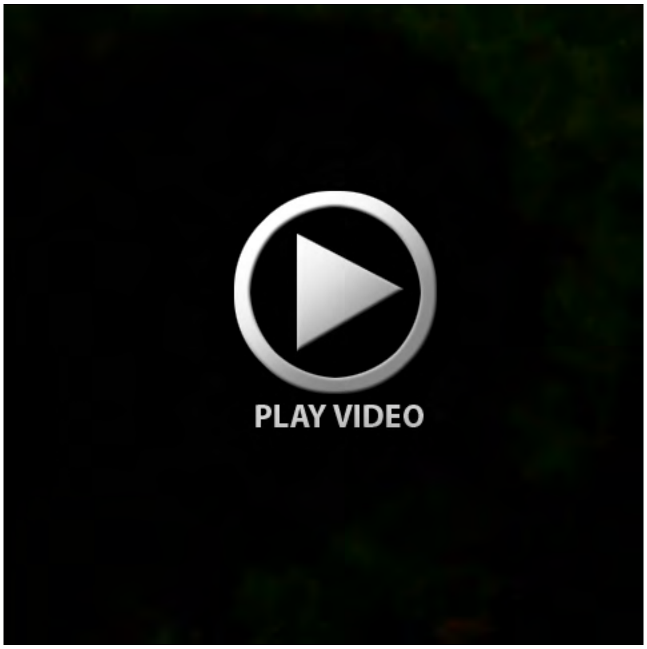

Movie 1. LC8 localises to nodal cilia. A 3 somite stage mouse embryonic node, stained for anti acetylated tubulin (red) and LC8 (green). A confocal image stack allowing all cilia within the node to be visualised, revealing that LC8 staining is evident in all nodal cilia.

Table S1. Outflow tract

Phenotype	C3.C-*Atmin*$^{gpg6/gpg6}$		*Dynll1*$^{GT/GT}$
Outflow tract – Normal	1/25	(4%)	0 (0%)
Outflow tract – IAA	12/25	(48%)	14 (67%)
Outflow tract - Common trunk	9/25	(36%)	7 (33%)
Outflow tract - Restricted aortic arch	3/25	(12%)	0 (0%)
